# Unveiling the Origin of Morphological Instability in Topologically Complex Electrocatalytic Nanostructures

**DOI:** 10.1021/jacs.5c07842

**Published:** 2025-09-06

**Authors:** Yawei Li, James L. Hart, Ramchandra Gawas, Zhiyong Xia, Pietro P. Lopes, Jieyu Zhang, Siming Li, Yucheng Wang, Mitra Taheri, Ian McCue, Joshua Snyder

**Affiliations:** † School of Chemistry and Chemical Engineering, 12441 Shanxi University, Taiyuan 030006, China; ‡ Department of Chemical Engineering, 6527 Drexel University, Philadelphia, Pennsylvania 19104, United States; § Department of Material Science and Engineering, Drexel University, Philadelphia, Pennsylvania 19014, United States; ∥ Department of Materials Science and Engineering, 1466 Johns Hopkins University, Baltimore, Maryland 21218, United States; ⊥ Johns Hopkins University Applied Physics Laboratory, Laurel, Maryland 20723, United States; # Materials Science Division, 1291 Argonne National Lab, Lemont, Illinois 60439, United States; ¶ College of Chemistry and Chemical Engineering, 12466 Xiamen University, Xiamen 361005, China; ∇ Department of Materials Science and Engineering, 3270 Northwestern University, Easton, Evanston, Illinois 60208, United States

## Abstract

Coarsening and degradation phenomena in metals have largely focused on thermally driven processes, such as bulk and surface diffusion. However, dramatic coarsening has been reported in high-surface-area, nanometer-sized Pt-based catalysts during potential cycling in an electrolyte at room temperature—a temperature too low for the process to be explained purely by surface mobility values measured in both vacuum and electrolytes (∼10^–22^ and ∼10^–18^ cm^2^/s, respectively). This morphological evolution must be due to a different mechanism for mass transport that is sensitive to electrochemical conditions (e.g., electrolyte composition, potential limits, and scan rate). However, there have been no notable studies of electrochemically induced coarsening in nanometer-sized electrocatalysts. Here, we unveil the origins of coarsening in an electrolyte through coupled in situ experiments and atomistic kinetic Monte Carlo (kMC) simulations. Our work demonstrates electrochemical coarsening is driven by two concurrent mechanisms that can be explained at the atomistic level: (i) dissolution/redeposition during the reduction of an oxidized species and (ii) rapid surface diffusion of undercoordinated atoms.

## Graphical abstract

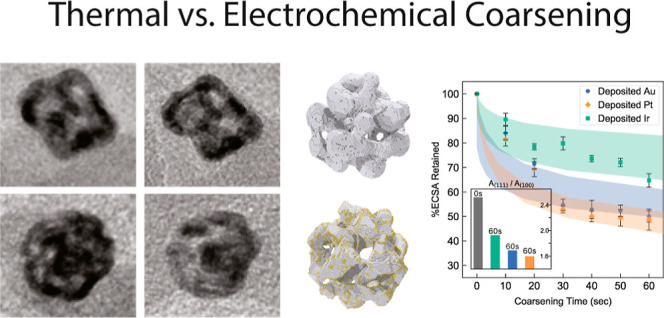

## Introduction

Electrochemical energy conversion technologies, such as electrolyzers and fuel cells, are critical to a renewable carbon-neutral energy portfolio.^1,2^ The growth of their integration into consumer and industrial applications is directly tied to economic descriptors that depend on their efficiency and operational lifetimes, ultimately determined by the performance of the electrocatalysts that make up the anodic and cathodic electrodes.^3,4^ While significant research efforts have been underway to develop earth-abundant electrocatalytic materials,^5^ necessary performance metrics, such as energy density and energy efficiency, have only reliably been met with platinum group metal (PGM)-incorporated materials.^6,7^ However, the high cost and relative scarcity of PGM necessitate significant improvements in their active area and mass-normalized activities to lower their mass loadings.^1,8–10^ For example, the widespread commercialization of polymer electrolyte membrane fuel cells remains limited by the high loading of platinum (Pt) to compensate for the slow oxygen reduction reaction (ORR) kinetics.^11,12^

To overcome these limitations, recent electrocatalyst development has focused on the design and synthesis of nanoarchitectured catalysts (NACs).^13–31^ We define NACs as a broad class of catalysts—consisting of tailored surface facet ratios, feature sizes, surface site distributions, and composition—where the activity and surface fraction of catalytically useful precious metal have been maximized.^1^ NACs possess higher surface-area-to-volume ratios than spherical nanoparticles due to more complex shapes—such as nanocages,^14,15^ porous networks,^16–19^ and jagged nanowires^20,32^ —and as a result have continuously set new records for specific and mass activities over the last ten years. These catalysts possess activities 4–20× over the industry standard Pt/C but suffer electrochemical active surface area (ECSA) losses of 8–40% during accelerated stability testing (AST), as shown in Figure 1.^14,15,20,22,33–59^ Perhaps more concerning, these ECSA losses become more severe with increasing upper potential limit (UPL) during load cycling.^33,49,55^

**Figure 1:**
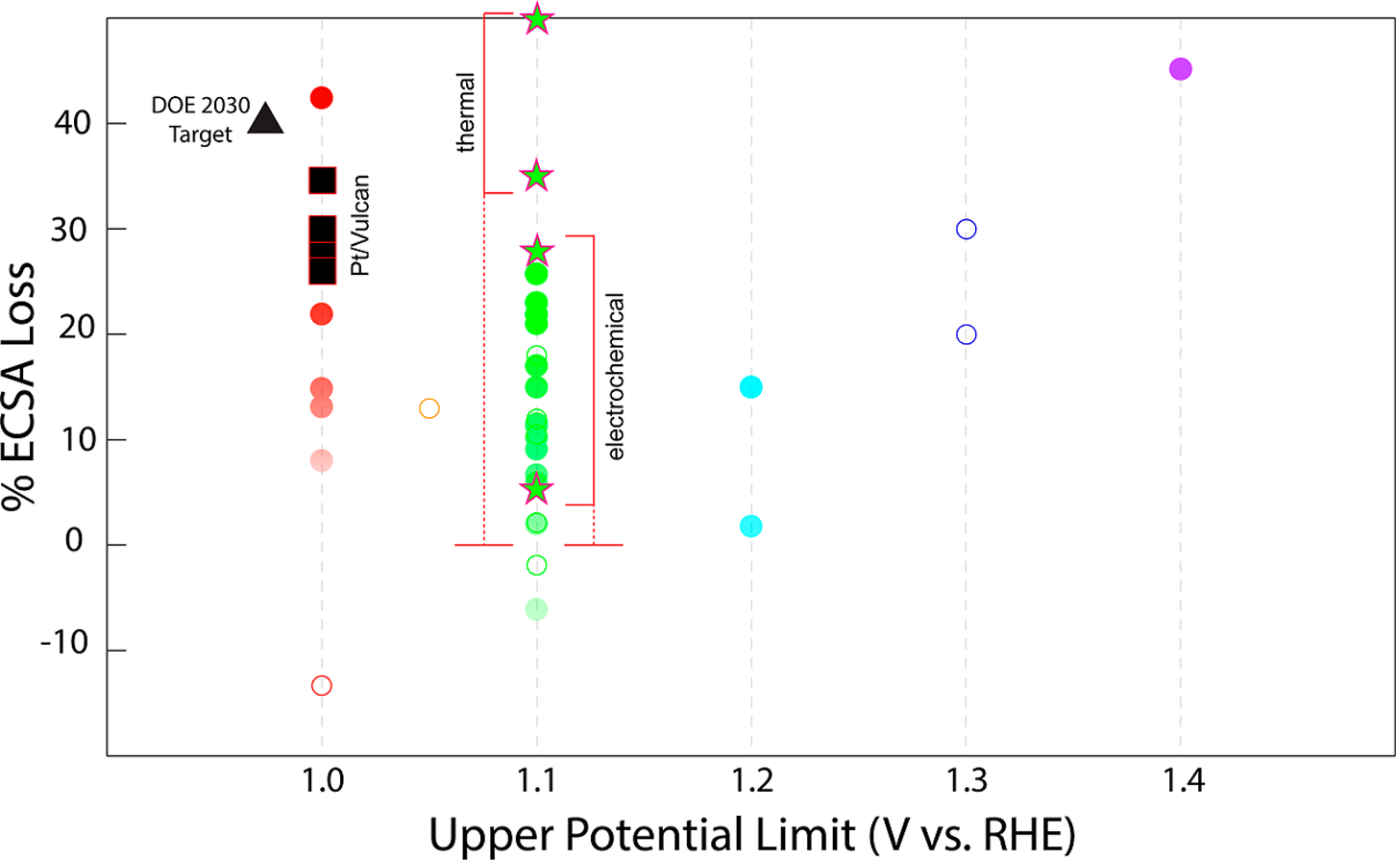
Summary of representative studies on ECSA change with various upper potential limits after AST for nanoarchitectured (open-frameworks, nanowires, anisotropic shapes, etc.) Pt-based electrocatalysts.^14,15,22,33–59^ Solid circles represent AST protocols of 10,000 cycles, and open circles are for protocols with a number of cycles other than 10,000. Green stars represent data from this article for both electrochemically and thermally driven coarsening. The DOE 2030 target^60^ for ECSA retention (90,000 cycles) (black triangle) and representative Pt/Vulcan data from literature (black squares)^61–64^ are also included.

This ECSA degradation is distinctly different from the activity loss observed in traditional spherical nanoparticle electrocatalysts during load cycling. For instance, carbon-supported solid Pt and Pt-alloy (both homogeneous and core–shell^65,66^ ) nanoparticle electrocatalysts under load cycling have two primary active area loss mechanisms: (1) sintering/agglomeration due to weak physisorption onto the carbon support as well as oxidative loss of carbon support and (2) Ostwald ripening driven by the dissolution of the metallic catalyst.^67^ In contrast, NACs have been shown to degrade (i.e., lose active area per mass) without significant mass exchange between particles.^27,31,40,49^ Thus, unveiling the atomic-scale processes that govern the electrochemical degradation of these nanostructured materials at the fundamental level is critical for the development of next-generation electrocatalysts that are active and stable.

In this study, we examine the coarsening behavior of a model NAC material and highlight the limiting atomic processes that govern changes in the surface area and facet ratios during electrochemical cycling and thermal annealing. Through the combination of experimental and computational metrics, we demonstrate that surface dopants with either high reduction potentials or low surface mobility can limit the loss in the total electrochemically active surface area over an AST by a factor of 5. This work is the first instance where electrochemical coarsening is deconvoluted into distinct surface diffusion and dissolution/redeposition events. Insights from this work will have a measurable impact on the integration of active and morphologically stable materials into electrochemical energy conversion and storage devices.

## Results and Discussion

We use nanoporous NiPt nanoparticles (np-NiPt) as representative NACs to assess morphological evolution (defined as changes in surface area, surface facet ratios, composition, and concentration of surface sites) during load cycling. NACs, especially nanoporous metals, have intrinsically metastable morphologies, which provide a large thermodynamic driving force to reduce their surface energy and thus surface area.^49,68^ We fabricate np-NiPt nanoparticles by electrochemically dealloying precursor Ni_80_Pt_20_ alloy nanoparticles and then deposit Ir or Au on the surface of the nanoporous nanoparticles, Figure 2A,B (see Methods for further details). The resulting surfaces of the nanoparticles are uniformly decorated with a fraction of ∼5 atom % Au (np-NiPt + Au) and Ir (np-NiPt + Ir) (Figure 2C and Table S1), respectively.

**Figure 2:**
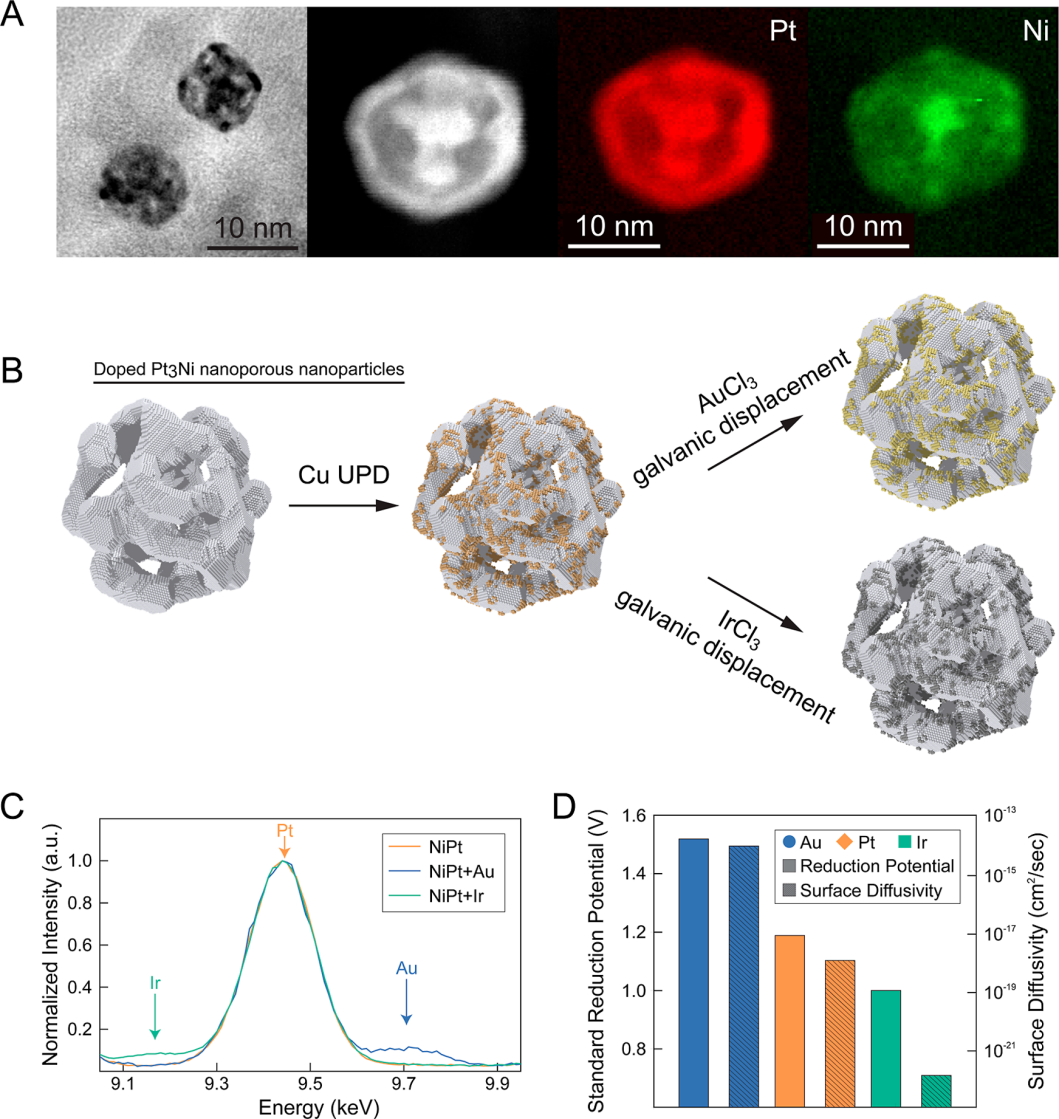
(A) TEM image, HAADF-STEM image, and corresponding Pt and Ni EELS mapping of nanoporous NiPt nanoparticles (np-NiPt) examined in this study. (B) Schematic illustration of the surface doping process. Cu is first underpotentially deposited onto np-NiPt nanoparticles and then decorated with either Au or Ir via galvanic displacement. (C) STEM-EDS analysis of the Ir, Pt, and Au L_α1_ peaks. (D) Reduction potential and surface diffusivity comparison between Au, Pt, and Ir.

The benefit of controlling the introduction of dopants is that the initial surface conditions are identical for all elemental additions. Au and Ir are chosen for both their differing surface mobilities and standard reduction potentials, as demonstrated in Figure 2D. Surface diffusion rates for metallic species scale roughly with the melting point of the metal.^69,70^ As such, we expect Ir dopants to have a lower surface mobility than Pt atoms and Au dopants to have a higher surface mobility.^31,69^ The standard reduction potentials of Ir^3+^/Ir, Pt^2+^/Pt, and Au^3+^/Au are 1.0,^71^ 1.188,^72^ and 1.52 ^73^ V vs SHE, respectively. For both surface mobility and standard reduction potential, Pt values sit about halfway between those of Ir and Au. The expected result is an impact on both the mobility of surface atoms and the average “nobility” of the surface following dopant incorporation as a function of dopant identity.

To characterize the morphological evolution of np-NiPt during thermal coarsening, where morphology evolution is limited to temperature-induced surface mobility, we applied in situ heating transmission electron microscopy (TEM), shown in Figure 3A. Starting from room temperature, we do not observe substantial morphological changes until about 300 °C, and the nanoporous structure coarsens into a solid particle after holding at 450 °C for 10 min. In situ heating TEM of np-NiPt + Ir and np-NiPt + Au (Figure 3A) nanoparticles under identical thermal conditions yields distinctly different time-dependent morphology profiles. In the case of capillary-driven coarsening, morphological evolution will be limited by the slowest diffusing species.^74^ For nanoporous metals, it has been demonstrated that the rate-limiting behavior is step-edge flow.^75^ Given Au’s high surface mobility, we expect Pt diffusion to be the rate-limiting behavior for both the np-NiPt and np-NiPt + Au samples, and both compositions will exhibit similar degrees of coarsening. However, the presence of Ir, likely concentrated at step edges,^31,76^ will substantially reduce the surface mobility and lead to a negligible change in nanoporous morphology, confirmed in Figures 3A and 3C.

**Figure 3:**
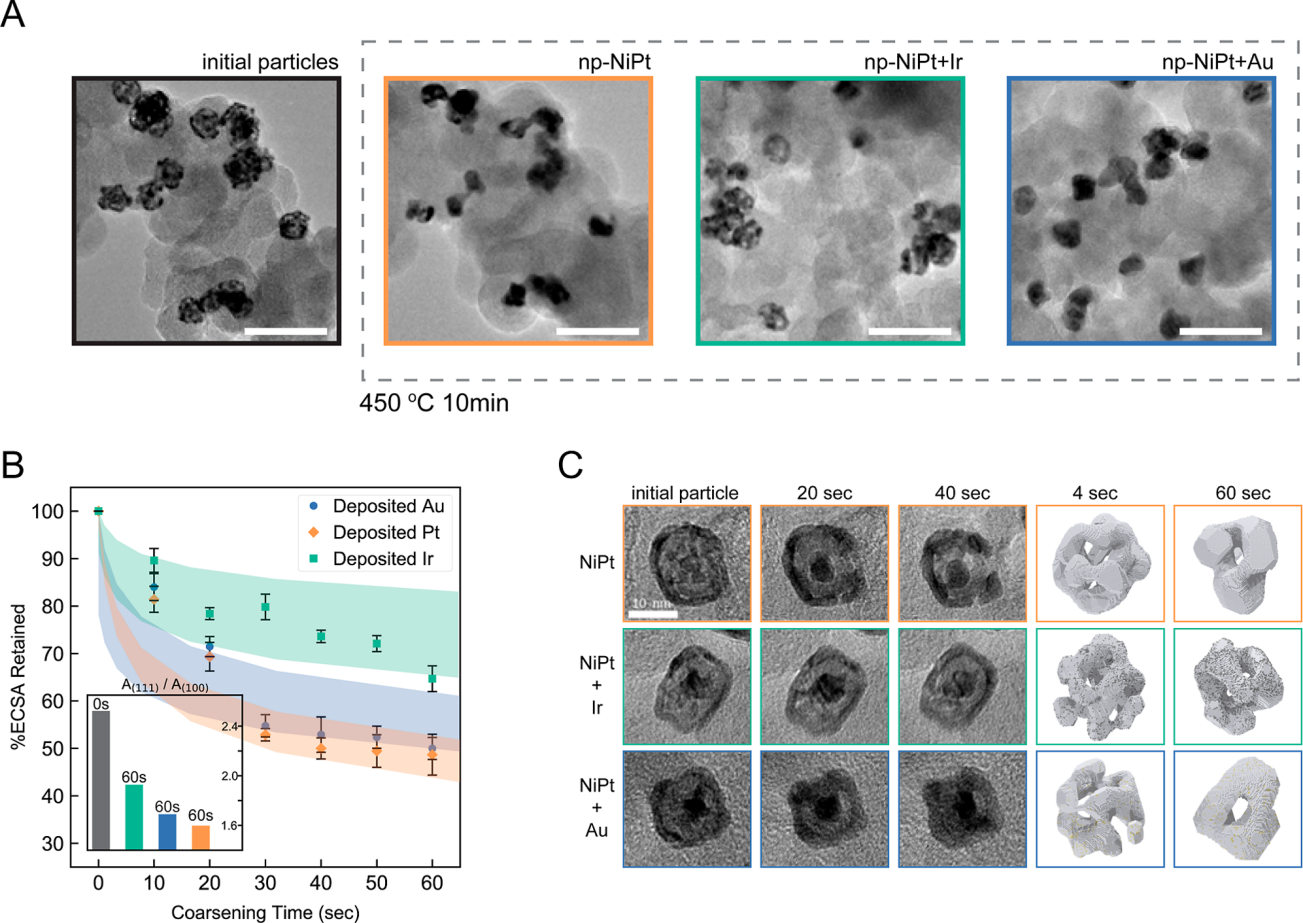
(A) Thermal coarsening of np-NiPt, np-NiPt + Au, and np-NiPt + Pt nanoparticles through in situ heating TEM at 450 °C for 10 min. The scale bar is 50 nm. (B) Experimental summary (markers) of ECSA as a function of time for np-NiPt + Au, np-NiPt, and np-NiPt + Ir at a constant temperature of 450 °C; kMC simulation results (colored bands) were incorporated where the upper bound refers to total surface area and the lower bound refers to active surface area. The inset shows the change in the ratio of 111/100 facet area after 60 simulated seconds with the indicated surface decoration. (C) In situ heating TEM series of np-NiPt, np-NiPt + Ir, and np-NiPt + Au at a temperature of 450 °C (left) and corresponding kMC simulations of thermal coarsening (right) after the indicated time duration.

Figure 3B contains a summary of time-dependent ECSA for np-NiPt, np-NiPt + Ir, and np-NiPt + Au at 450 °C. For these experiments, catalyst films on glassy carbon (GC) substrates are exposed to elevated temperatures in a furnace under an inert environment for a fixed time. The ECSA is then measured electrochemically (CO stripping) in a hanging meniscus rotating disk electrode (RDE) configuration (details in the Methods section). In agreement with our assessment, the ECSA data indicate that a partial monolayer (ML) of Au has a negligible effect on thermal coarsening, but the partial ML of Ir helps retain much of the high aspect ratio morphology and area (Figure 3B,C). This morphological trend for each dopant is mirrored in our atomistic kinetic Monte Carlo (kMC) simulations of thermal coarsening in nanoporous nanoparticles via surface diffusion, as shown in Figures 3B and 3C. While the total surface area of the simulated nanoparticles (lower bound, Figure 3B) decreases more than in the experiments, the estimated active surface area (upper bound, Figure 3B), which accounts for changes in facet distribution, decreases less than in the experiments; we expect the experimentally measured ECSA to fall between these boundaries. In agreement with our above assessment, we find that the slow-diffusing Ir atoms collect at step edges (Figures S3 and S4) and inhibit mass transport. In contrast, fast-diffusing Au atoms do not impact Pt transport across the surface.

We now turn our attention to coarsening in electrochemical environments during an AST protocol with an UPL of 1.1 V vs reversible hydrogen electrode (RHE) at room temperature (Figure 4). Similar to thermal coarsening, the presence of an Ir partial ML results in a negligible morphological change after AST in an electrolyte. We note that at the UPLs used, surface Ir is likely to be oxidized to higher-valent IrO_*x*_ species.^77^ As transition metal oxides possess lower surface mobility than their constituent metals,^78^ this does not change our analysis or conclusions as in all cases the Ir-based component, regardless of its oxidation state, is the slowest-moving component. IrO_*x*_ species are also sufficiently stable at the potentials tested here.^77^ However, unlike the thermal environment, electrochemical coarsening is also inhibited by the presence of a partial ML of Au, as shown in Figures 4 and 5. It has been previously demonstrated that Au on or near a Pt surface increases the “nobility” of neighboring Pt atoms,^65^ which we expect to limit the degree of Pt dissolution during a full redox cycle by increasing the onset potential for Pt oxidation.^79,80^ The stabilizing effect of Au during electrochemical coarsening suggests that under electrochemical cycling there must be an additional mechanism for mass transport: Pt dissolution followed by redeposition.

**Figure 4:**
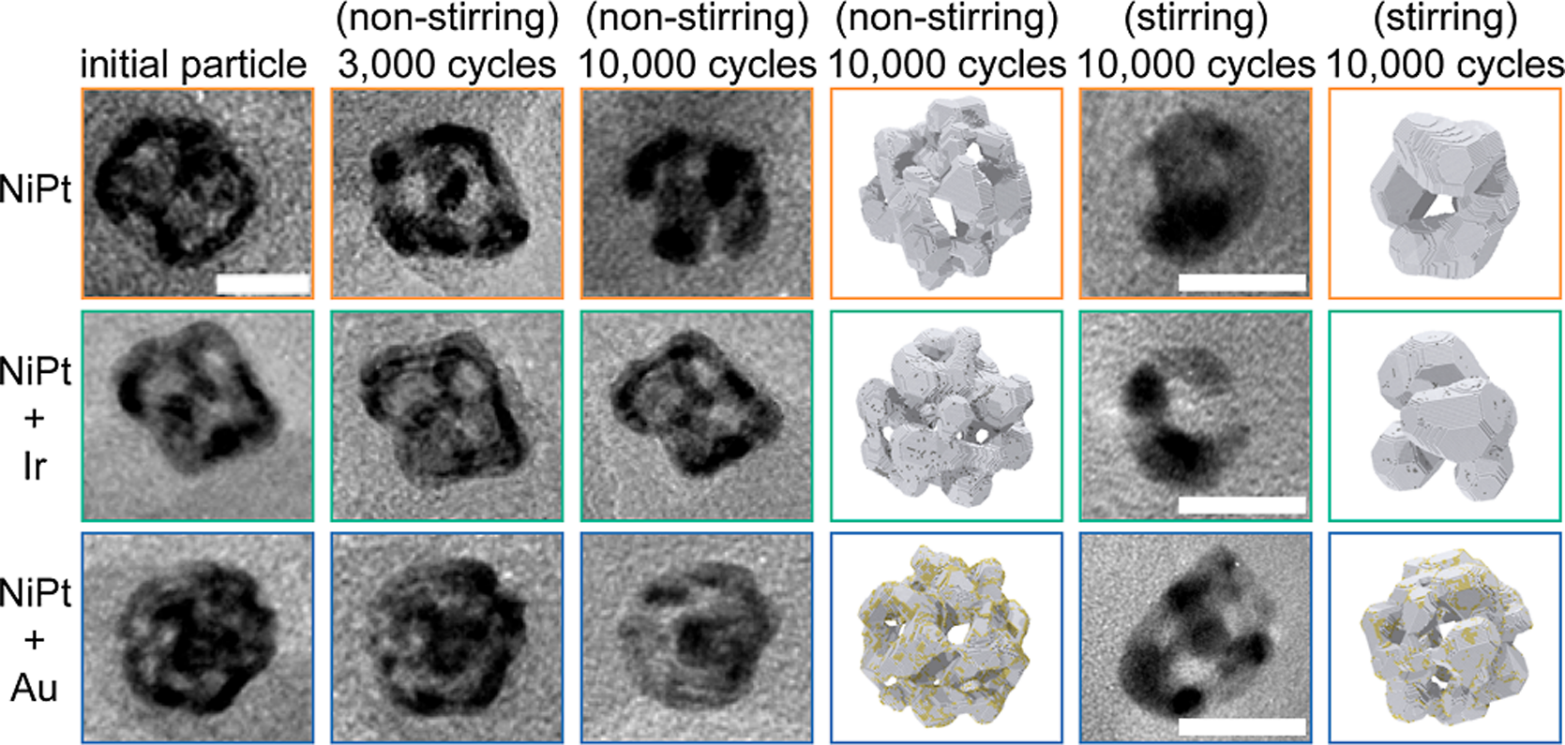
(Left) Identical location (IL)-TEM series and corresponding kMC simulation (with dissolution/redeposition integrated into the coarsening mechanism) of np-NiPt, np-NiPt + Ir, and np-NiPt + Au during AST in Ar-purged 0.1 M HClO_4_ at room temperature between 0.6 and 1.1 V vs RHE with a sweep rate of 50 mV s^–1^. The electrolyte is static. The scale bar is 10 nm. (Right) IL-TEM and corresponding kMC simulation of np-NiPt, np-NiPt + Ir, and np-NiPt + Au after 10,000 cycles with a stirred electrolyte to induce Pt^2+^ ion concentration gradients.

**Figure 5:**
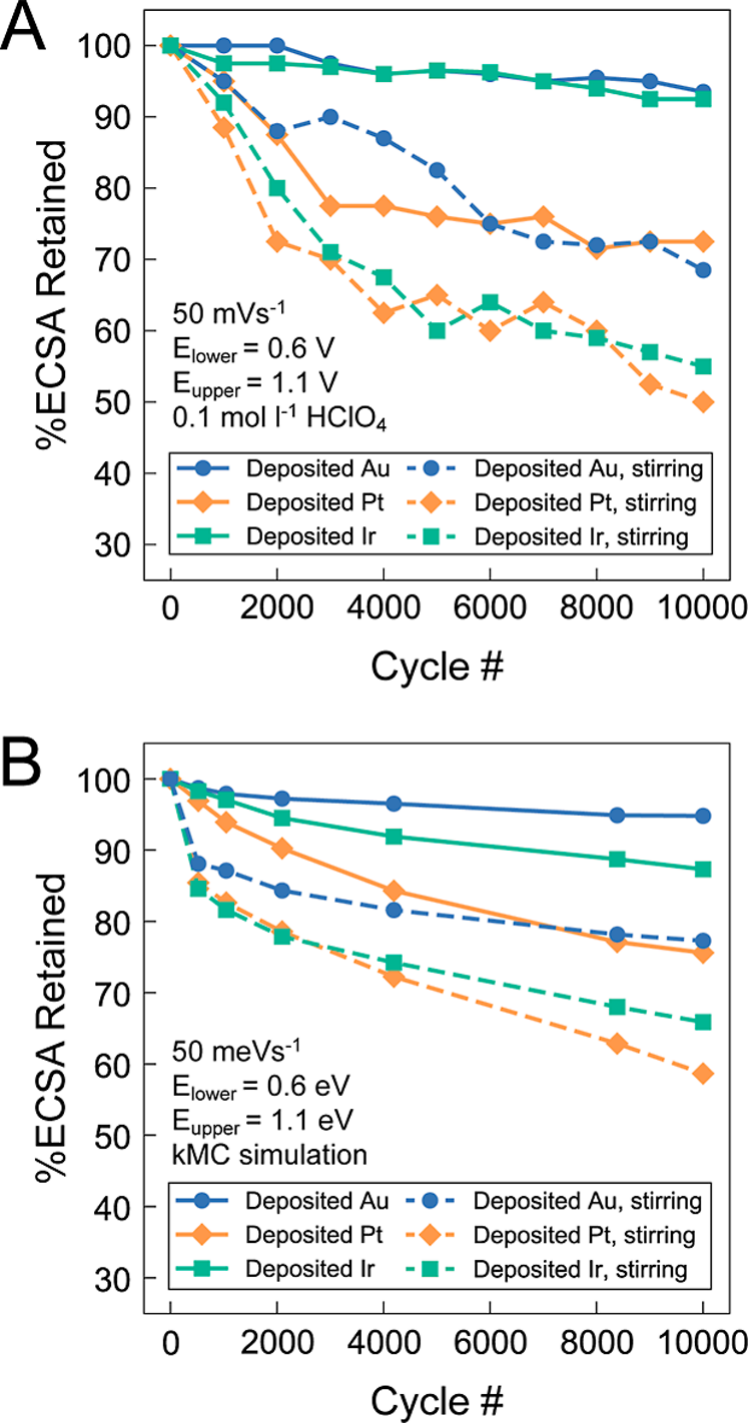
(A) Experimental and (B) kMC-simulated percent ECSA retained as a function of AST cycle number for np-NiPt + Au, np-NiPt, and np-NiPt + Ir with (dashed line) and without (solid line) electrolyte stirring. AST was assessed in Ar-purged 0.1 M HClO_4_ at room temperature between 0.6 and 1.1 V vs RHE with a sweep rate of 50 mV s^–1^.

This mechanism is distinct from Ostwald ripening because mass is not transferred between individual particles. Unlike solid nanoparticles, NACs have local regions of both positive and negative curvature and a highly tortuous morphology, which inhibits mass transport between particles until they coarsen and their morphologies resemble that of a dense nanoparticle.^81–84^ Prior to this shape transition, coarsening is confined to individual NAC particles, by which material will dissolve from regions of high positive curvature (during an anodic/cathodic potential sweeps^65,85^ ) and be redistributed along the surface via electrodeposition during the cathodic sweep. This event will couple with surface diffusion and result in the coarsening of the porous structure. Furthermore, any formation and reduction of surface oxides will lead to a disordered surface structure with a high density of adatoms, which will rapidly incorporate into the nearest step edge to reduce the energy of the system.^86,87^ The experimental degradation trends are confirmed in the kMC simulations by implementing a simple dissolution/redeposition mechanism using an atomistic description of the Butler–Volmer equation (details in the Methods section). Without this mechanism, a model driven purely by surface diffusion exhibits limited coarsening in an electrolyte at RT for np-NiPt nanoparticles.

What accentuates dissolution and redeposition in NACs is their tortuosity: the interconnected solid network increases the probability of a cationic species contacting the surface prior to elution into the bulk electrolyte. To further demonstrate the impact of dissolution/redeposition, we compared the ECSA retained during AST with and without convective stirring in the electrolyte, shown in Figures 4, 5A, and 5B. The stirring condition is analogous to that of a fast-flowing electrolyte, which will ensure that a Pt ion concentration gradient is established radially in the NAC and increase the probability of Pt ion elution into the bulk solution. This will also potentially increase the diffusion distance along the surface prior to redeposition. There is a substantial difference for both experimental and simulated coarsening, shown in Figures 5A and 5B, across all three compositions with and without convection. The undoped np-NiPt and np-NiPt + Ir samples behave in a similar manner, showing a much higher ECSA loss with convection in comparison to that for np-NiPt + Au. The origin of these differing responses is associated with the dopant metal-induced changes in the oxophilicity of the surface, which directly impacts the degree of surface oxidation and consequently the amount of solubilized Pt ions formed during a potential sweep.

In the absence of convective Pt ion removal, dissolution/redeposition during repetitive redox cycles introduces low-coordinated surface atoms, which then undergo surface diffusion^88^ in an analogous mechanism to porosity evolution during dealloying.^68^ The relative concentration of these surface defects (e.g., adatoms versus kink atoms) will be dependent on the UPL, potential sweep rate, and the load cycling profile (e.g., a triangular versus square wave^88^). A summary of our proposed mechanisms is listed in Figure 6. Au surface atoms reduce the oxophilicity of neighboring Pt atoms, reducing the quantity of locally oxidized and subsequently dissolved Pt per potential cycle. In addition to electronic effects, Au at the step edges could also decrease the degree of Pt oxidation/dissolution per cycle by passivation of the lower coordinated Pt atoms.

**Figure 6:**
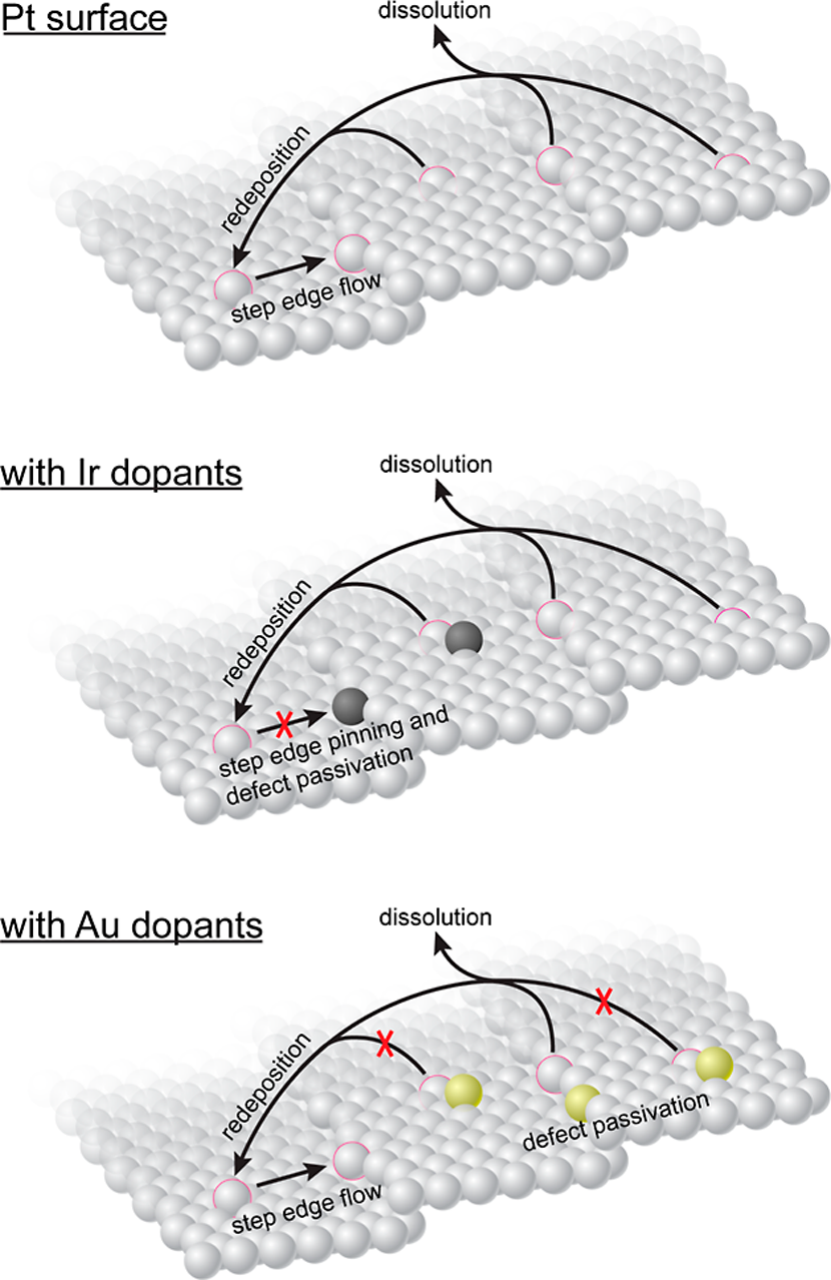
Illustration demonstrating the key differences between Au and Ir dopants on electrochemical coarsening: Au dopants decrease the frequency of Pt dissolution events, whereas Ir dopants decrease the surface mobility of Pt atoms.

Our kMC simulations, Figure S3, demonstrate the Au-doped particles have a higher probability for the dopant to be preferentially located at step and edge sites. Both the decrease in the degree of Pt surface oxidation, corresponding to a lower degree of roughening following reduction, and the subsequent reduction in Pt elution and redeposition reduce step-edge flow during cycling. Convection increases the diffusion distance, but the frequency of dissolution/redeposition events is lower than that of undoped np-NiPt. The behavior of Ir dopants is more nuanced in that Ir is not expected to affect the propensity of neighboring Pt atoms to dissolve. However, Ir dopants are expected to pin rate-limiting sites for coarsening (here, steps^31,76^ ), slowing surface diffusion and potentially protecting lower coordinated Pt atoms.

The reduction in surface mobility afforded by Ir, however, becomes negligible under convective conditions when mass can be transported across the surface via nonsurface diffusional processes and material can be lost to the bulk solution. This observation explains why Ir dopants have no substantial effect on electrochemical coarsening under stirring conditions, whereas Au dopants are protective under both static and convective electrochemical conditions. As noted in the beginning, the morphology of nanoporous metals affords intrinsic protection to this electrochemical degradation mechanism: the tortuosity of the porous network makes it more challenging (compared to a thin film) for species to be swept into the electrolyte, and the high fraction of saddle points increases the average coordination number of surface atoms to prevent dissolution during potential cycling. Unfortunately, these morphological factors are universal across nanoporous metals and are thus challenging to probe in our study. However, this degradation mechanism is expected to be more substantial in NACs that primarily have positive curvature such as dense nanoparticles and nanowires.

To further characterize the impact of metal surface dopants on Pt dissolution, in situ ICP-MS is used to simultaneously (i) measure the dissolution rate of Pt during potential cycling and (ii) confirm the suppression of Pt dissolution with the presence of partial ML of Au, Figures 7A and 7B. Electrolyte flow is used to acquire the in situ sample and transfer it to the ICP-MS, mimicking the convective conditions. The mass loss of Pt during a single cathodic–anodic redox cycle is found to decrease with increasing Au coverage (Figure 7A and 7B). As has been demonstrated previously,^65,89^ the majority of the Pt dissolution occurs during the cathodic sweep through reduction of the oxide. Figures 7C and 7D show in situ ICP-MS measurements of Pt dissolution rates from np-NiPt, np-NiPt + Ir, and np-NiPt + Au. There is a clear reduction in the rate of Pt dissolution, during the cathodic sweep, in the presence of the Ir and Au dopants. The reduction in Pt dissolution with Ir is lower than that observed for Au. The partial ML of Ir can passivate some of the low-coordinated steps, but not all, while the same coverage of Au can both passivate and enhance the nobility of neighboring exposed Pt atoms, which results in a smaller amount of Pt dissolved. This is supported by the positive shift in the onset of Pt dissolution for the np-NiPt + Au in comparison to np-NiPt and np-NiPt + Ir, Figure 7D. Previous work has shown that subsurface Au stabilizes surface Pt through electronic and structural modification; this is in addition to the stabilization of lower coordinated Pt atoms through passivation by surface Au atoms.^65,90^ Additionally, we also see a larger degree of Ir dissolution at the higher UPLs in comparison to Au, which could influence the comparative result, as shown in Figure S6. The total amount of metal ions lost to the bulk solution during a full AST protocol (10,000 cycles) is also quantified through inductively coupled plasma optical emission spectroscopy (ICP-OES) analysis of the electrolyte post-AST (Figure S7). After 10,000 AST potential cycles, both Pt and Ni losses are detected for all cases.

**Figure 7:**
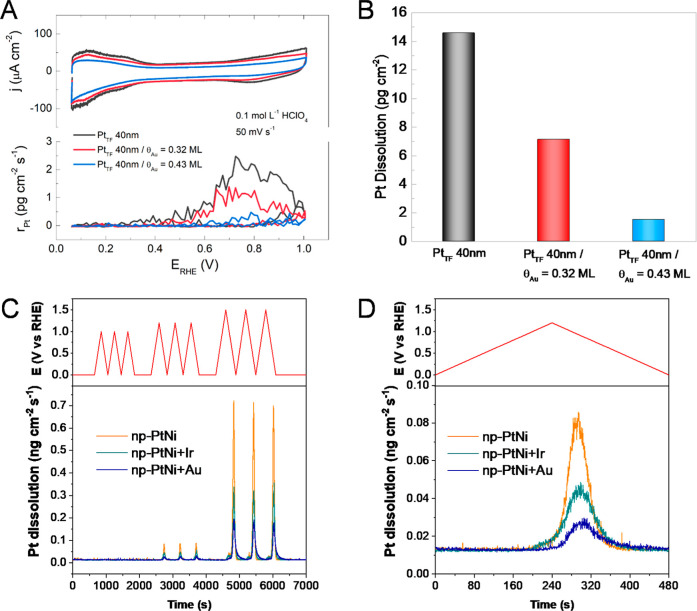
(A) Cyclic voltammetry curves (top) and corresponding in situ Pt dissolution rates (bottom) for RF sputter-deposited 40 nm Pt thin film on glassy carbon (black) and partially covered with a Au overlayer with varying coverage (Θ_Au_ = 0.32 ML in red and Θ_Au_ = 0.43 ML in blue). (B) Comparison of Pt dissolution for all three films. (C) Pt dissolution rates for nanoporous nanoparticles (np-NiPt (orange), np-NiPt + Ir (green), and np-NiPt + Au (blue)) during triangular wave potential cycling for three upper potential limits 1.0, 1.2, and 1.5 V vs RHE. (D) Higher-resolution view of Pt dissolution rates with a cycling upper potential limit of 1.2 V vs RHE. All experiments were performed in 0.1 M HClO_4_ and a voltammetry sweep rate of 50 mV s^–1^ at room temperature.

Surface and subsurface Ni lost during AST are mainly due to Pt transport (dissolution or surface diffusion) and exposure of subsurface Ni as the structure coarsens. In the presence of partial monolayers of both Ir and Au, the total loss of Ni and Pt to the solution decreases. This is not unexpected as both Au and Ir slow coarsening, which reduces surface mobility-driven exposure of underlying Ni, limiting its dissolution. The tangible impact of subsurface Ni loss is a decay in the intrinsic activity of the catalyst (Figure S8). In the presence of Ir dopants, there is no loss in specific activity (SA) as the Ir dopant acts to retain subsurface Ni and its impact on the ORR activity. The SA for np-NiPt + Ir even improves over the course of the ORR, likely due to either surface alloying of Ir with Pt or redistribution of Ir on the surface, increasing exposure of the more reactive Pt. The opposite is observed for the Au dopant, Figures S5 and S8. As the NAC coarsens during the AST, Au will remain on the surface as the UPL is below the potential for significant Au oxidation/dissolution,^90^ see Figure S6. The result will be an increasing surface coverage of Au as the particle surface area decreases, blocking an increasing fraction of the surface with inactive Au and leading to reduced ORR activity. Similar effects have been observed with Au coatings on Pt thin films, where ORR performance significantly reduced beyond a Au surface coverage of 0.2 ML^65^ and in PtNiAu alloy nanoparticles beyond a Au content of 3 at. %.^91^ The increase in the onset potential for CO stripping, Figure S5, following the AST protocol is also indicative of a surface smoothening and passivation by inactive species, i.e., Au. High-resolution TEM with EDS mapping, Figures S9 and S10, of the beginning of life (BOL) and end of life (EOL) shows significant aggregation of Au for np-NiPt + Au and a moderate loss of Ir for np-NiPt + Ir. These in situ ICP-MS results provide further support for the link between electrochemical coarsening and Pt dissolution/redeposition.

## Conclusions

For the first time, we effectively deconvoluted the distinct surface diffusion and dissolution atomic processes that govern electrochemical coarsening. Contrary to current strategies, future catalysts need to be designed to inhibit both Pt dissolution and mass transport across multiple length scales (from within the electrode structure to within the nanoporous particle itself). In this regard, a new challenge emerges: balancing a high ECSA without negatively impacting the intrinsic activity. Au and Ir are used here for demonstrative and investigative purposes, and their use in commercial catalyst materials must be judiciously evaluated by considering their cost and the negative impact on ORR activity. The most promising dopants are likely elements that are known to form stable, inert oxide species at high UPLs (e.g., Ti or Ta); these elements could be beneficial from a surface-mobility perspective (as compounds with high cohesive energies will have sluggish diffusion), but first-principles calculations would be necessary to screen their effect on the electrochemical stability of adjacent Pt species. In addition, these elements are challenging to electrodeposit at room temperature in aqueous electrolytes and would thus require specialized approaches in bespoke ionic liquids. Ultimately, the development and integration of these morphologically stable NACs will yield significant improvements in both the precious metal utilization and operational longevity for electrochemical energy conversion and storage devices.

## Methods

### Nanoparticle and Catalyst Synthesis

The precursor Ni_80_Pt_20_ alloy nanoparticles were synthesized through an organic solvothermal reduction method.^23,31^ Ni­(acac)_2_ (0.80 mmol), oleylamine (4 mL), and 1,2-tetradecadeniol (TDD, 0.5 mmol) were initially introduced into 10 mL of diphenyl ether (DPE) at 100 °C and then heated to 180 °C under an Ar atmosphere. Following several vacuum and Ar purging cycles, a solution of 0.20 mmol Pt­(acac)_2_ and 3 mmol adamantanecarboxylic acid (ACA) in 3 mL of dichlorobenzene (DCB) was injected into the stirring solution at 180 °C, and then the solution was heated to 225 °C. After the temperature remained for 1 h at 225 °C, the solution was cooled to room temperature under flowing Ar. The formed nanoparticles were centrifuged at 8000 rpm, washed with hexane/ethanol, and finally deposited onto carbon support (Vulcan XC-72) through a colloidal deposition process. After centrifugation and three washing cycles with hexane/ethanol, the as-made catalyst was annealed in a tube furnace at 180 °C in air for 1 h, followed by 400 °C in H_2_/Ar for 2 h. The metal loading, determined through thermogravimetric analysis (TGA), was found to be 20 wt % metal on carbon for the as-synthesized Ni_80_Pt_20_ alloy particles and 13 wt % metal on carbon for the dealloyed np-NiPt/C catalyst.

### Dealloying and Electrochemical Measurements

The as-annealed nanoparticle catalysts were dealloyed, electrochemically characterized, aged, and assessed for oxygen reduction reaction (ORR) activity in a three-electrode cell using a rotating disk electrode (RDE) setup from Pine Instruments controlled by a Metrohm Autolab potentiostat (PGSTAT302N). The counter electrode was Pt mesh (99.9%, Alfa Aesar) bonded to the end of a Pt wire (99.9%, Alfa Aesar). The Ag/AgCl (BASi) reference electrode was calibrated against a hydrogen reference and found to have an offset of 0.27 V at 25 °C for 0.1 M HClO_4_. All potentials listed are referenced to the reversible hydrogen electrode (RHE). Prior to any electrochemical experiments, all glassware was cleaned by soaking in a solution of concentrated 1:1 H_2_SO_4_/HNO_3_ for at least 8 h, followed by rinsing and boiling in Millipore (Milli-Q Synthesis A10) water for 3 times.

The thin-film catalyst layers, with 15 μg_Pt_ cm^–2^ loading, were formed on glassy carbon (GC) disks (5 mm diameter, 0.196 cm^2^) by drop casting from a catalyst ink and drying under a flow of Ar. The catalyst ink was composed of a 4:1 H_2_O/IPA volume ratio solvent solution with a solid catalyst concentration of 1 mg_catalyst_ mL^–1^. Additionally, 0.5 μL of Nafion 5 wt % solution (Ion Power LQ-1105 1100 EW) per milligram of catalyst was added to the ink (ionomer/carbon mass ratio ≈1:37.2) to aid in dispersion and adhesion of the catalyst particles to the GC substrate.

Electrochemical dealloying of catalysts, to form np-Ni_30_Pt_70_, was accomplished in Ar-purged 0.1 M HClO_4_ in a standard three-electrode electrochemical cell by cycling the potential between 0.05 and 1.2 V vs RHE at 250 mV s^–1^ for 65 cycles. The accelerated stability test (AST) consisted of 10,000 triangular wave potential cycles between two specified potential limits in 200 mL of Ar-purged 0.1 M HClO_4_ with a sweep rate of 50 mV s^–1^ at room temperature. The starting composition of np-NiPt is confirmed by inductively coupled plasma atomic emission spectroscopy (ICP-OES) and energy-dispersive X-ray spectroscopy (EDS) analysis. The evolution of the catalyst ECSA was determined through the integration of the current in the hydrogen underpotential deposition (*H*_UPD_) region of the CV curves and the current in the CO oxidation region of the CO stripping curve following the procedure outlined by van der Vliet et al.^92^ For the ORR activity measurement, the dealloyed catalyst, either before or after AST, was transferred to a three-electrode electrochemical cell containing fresh O_2_-saturated 0.1 M HClO_4_ at 25 °C. Anodic ORR polarization curves were recorded at 1600 rpm while running linear sweep voltammetry between 0.1 and 1.1 V vs RHE at 20 mV s^–1^. Experiments were repeated more than three times in order to confirm that the results were repeatable. Kinetic current densities for ORR were calculated using the Koutecky–Levich equation to adjust for mass transport limitations:

1 $$\frac{1}{i}=\frac{1}{i_{k}}+\frac{1}{i_{d}}$$

where *i* is the measured current density, *i*_d_ is the diffusion-limited current density, and *i*_k_ is the kinetic current density. Specific activities were obtained through normalizing the *i*_k_ at 0.9 V versus RHE by Pt surface area. All potentials are corrected for *iR* drop within the electrochemical cell.

### Partial Monolayer of Ir/Au Deposition

Partial monolayers (ML) of Ir and Au were deposited on the surface of the np-NiPt nanoparticles through the surface-limited redox replacement.^31,93^ First, a partial ML of Cu was underpotentially deposited on the surface of the nanoporous nanoparticles in a 1 mM CuSO_4_ + 0.1 M H_2_SO_4_ solution at a constant potential of 0.30 V vs RHE. After formation of the partial Cu ML, the catalyst layer was immersed in a solution of 0.025 mM IrCl_3_ or AuCl_3_ at open-circuit potential to drive the galvanic displacement of Cu with Ir or Au, respectively. After galvanic displacement of Cu, the Ir- or Au-doped nanoporous nanoparticles were washed with DI water prior to further testing.

### Thermal Coarsening ECSA Measurement

A series of catalyst-coated GC disk electrodes was prepared using the same batch of catalyst ink, and each electrode was assigned for only one time point. Prior to thermal coarsening, electrodes were transferred into the quartz tube, which was then purged with Ar (10 psi pressure) at room temperature for 15 min to ensure complete removal of oxygen. Subsequently, the tube was quickly placed inside the tube furnace already heated to a constant temperature of 450 °C. After a certain period, the tube was quickly taken out and transferred into an ice bath to let it cool down for 10 min while Ar was purging continuously. Finally, the electrodes were installed into the hanging meniscus RDE configuration^94^ for further electrochemical ECSA measurements. The evolution of the catalyst ECSA under thermal conditions as a function of time was determined through the integration of the current in the *H*_UPD_ region. Five electrodes for each time point were tested to confirm that the results were repeatable.

### Electrolyte Stirring Setup for Electrochemical Coarsening

A magnetic stir bar with 1.27 cm length (Fisherbrand PTFE) was placed in a standard three-electrode electrochemical cell, which was on a magnetic-stirrer device (Fisherbrand). Standard AST was accomplished in 200 mL of Ar-purged 0.1 M HClO_4_ with a stir bar rotating at 400 rpm.

Reynolds number in a stirred vessel is defined as^95^

2 $$Re=\frac{\rho ND^{2}}{\mu }=\frac{ND^{2}}{\nu }$$

where ρ is the density of the electrolyte, *N* is the rotational speed, *D* is the diameter of the agitator, and μ and ν are the dynamic viscosity and the kinematic viscosity of the electrolyte, respectively. In our case, *N* is 41.8879 rad/s, *D* is 1.27 cm, and ν is 0.00893 cm^2^/s in 0.1 M HClO_4_.^96^ By taking account of these parameters, the Reynolds number can be approximately estimated to be 7565.62, which is higher than 4000, indicating a turbulent flow.

### Morphological and Compositional Characterization

Transmission electron microscopy (TEM) (JEOL JEM-2100) and scanning TEM (STEM) (JEOL JEM-2100F with a Schottky source) were performed at 200 keV to visually characterize the microstructure of the nanoparticles. Scanning TEM (STEM) electron energy loss spectroscopy (EELS) (JEOL 2100F with a Quantum Gatan Imaging Filter) was used to measure Ni and Pt fractions and generate elemental maps. STEM analysis was conducted with a probe size of approximately 1 nm and a high-angle annular dark field (ADF) detector with inner and outer detection semiangles of 27 and 54 mrad, respectively. The Ni and Pt atomic maps in Figure 1 of the main text were generated from Ni *L*-edge and Pt *M*-edge EELS measurements. The STEM EDS data was acquired with an Oxford X-Max^N^ 80T EDS system with an 80 mm^2^ SSD. The presented X-ray spectra area was spatially averaged across individual nanoparticles. The amount of metal, Pt and Ni, transferred to the electrolyte during AST through catalyst dissolution was quantified with post-mortem inductively coupled plasma atomic emission spectroscopy (ICP-OES) (Thermo Scientific iCAP 7000) testing of the electrolyte. Identical location TEM (IL-TEM)^31^ was used to qualitatively track the change in nanoporous nanoparticle morphology as a function of cycle number during AST. A gold TEM grid (Pacific Grid Tech) with a carbon supportive film was used as both the working electrode for AST and the material support for TEM analysis.

In situ heating TEM experiments were performed within a JEOL 2100F microscope, equipped with a Schottky source and a high-resolution pole piece with Cs = 1.0 mm. The TEM was operated in bright-field imaging mode. Annealing was performed with two holders, the Gatan 626 hot stage (main text Figure 3A) and the DENSsolutions Lightning D9+ sample holder, using heating-only nanochips. High-frame-rate data was collected during annealing using a Gatan K2 IS direct detection camera, operated in IS mode.

### In Situ ICP-MS Characterization

Thin metal films of Pt and Au were deposited by magnetron sputter deposition^97^ on a mirror-polished glassy carbon substrate (base vacuum 1 × 10^–9^ Torr). The deposition rate was calibrated by using a quartz-crystal microbalance. The deposition rate of Pt film was set to 0.3 Å s^–1^ for ∼30 s, creating a 5 nm average nanograin size. Deposition of the Au thin film was done at 0.75 Å s^–1^, exposing the glassy carbon surface during approximately 5–10 min, making a ∼20–40 nm thick film. Simultaneous electrochemical and metal dissolution rate measurements were done by attaching a stationary probe to the RDE (SPRDE method^98^), and the fraction of electrolyte pumped from the electrode surface was analyzed directly into an ICP-MS (PerkinElmer, NexION 350S). Plasma, auxiliary, and nebulization flow rates were 15.6 L min^–1^, 1 L min^–1^, and 1 L min^–1^, respectively, and plasma RF power was set to 1600 W. Pt (195 a.m.u) and Au (197 a.m.u) ions were simultaneously measured in the ICP-MS at a 4 points per second total, while the working electrode was controlled by a Metrohm Autolab potentiostat (PGSTAT302N). The electrodes were rotating at 100 rpm, with a reproducible SPRDE collection efficiency at 0.25.^98^

Operando measurement of Pt dissolution rates from the np-NiPt, np-NiPt + Ir, and np-NiPt + Au catalysts was performed with online ICP-MS coupled with a Teflon flow cell. During the ICP-MS test, the glassy carbon electrode with a catalyst loading of 0.612 mg/cm^2^ was immersed into the flowing electrolyte, a Au wire was used as the counter electrode, and SCE was used as the reference electrode. The Ar-saturated 0.1 M HClO_4_ solution was the electrolyte, with a flow rate of 0.085 mL/s. A CV protocol triggered the Pt dissolution at a sweep speed of 50 mV/s in the potential ranges of 0.0 ∼ 1.0 V, 0.0 ∼ 1.2 V, and 0.0 ∼ 1.5 V.

### Kinetic Monte Carlo (kMC) Simulation, Kinetic Rate Equations, and Model Parameters

We used a lattice-based kMC algorithm, MESOSIM, to simulate coarsening in np-NiPt nanoparticles. Our model is based on the approach laid out in ref 99, which uses a first nearest neighbor, bond-breaking model to capture the morphological evolution physics during dealloying and subsequent coarsening occurring over experimental time scales.

The time evolution of the system is governed by the kMC algorithm with the following steps:

(1) Tabulate all *N* possible transitions, each indexed by *i* and possessing a rate *k*_*i*._
(2) Calculate the cumulative function *K*_*i*_ = ∑_*j* = 1_^*i*^ *k*_*j*_ for *i* = *i*, ...*N*.
(3) Select a random number ζ = (0,1].
(4) Determine the event from *K*_*i*–1_ < *rK*_*N*_ ≤ *K*_*i*_, where *K*_*N*_ = ∑_*j* = 1_^*N*^ *k*_*j*_ = *k*_total_.
(5) Update the simulation time by the time interval Δ*t* = −ln­(ζ)/*k*_total_.
(6) Go to step 1.

Specifically, rates for surface diffusion and dissolution in our model are governed by the expressions *k*_diff_ = ν_1_ exp­[−*nE*_B_/*k*_B_ *T*] and *k*_diss_ = ν_2_ exp­[(−*nE*_B_ – ϕ)/(*k*_B_ *T*)], where ν_1_ and ν_2_ are attempt frequencies, *n* is the number of first nearest neighbors, *E*_B_ is the bond energy, ϕ is the applied potential, *T* is the temperature, and *k*_B_ is the Boltzmann constant. These kinetic expressions have been shown to accurately model dissolution current versus potential as well as coarsening behavior in nanoporous metals.^75,99–103^

In previous kMC studies on nanoporous gold (formed via dealloying Ag out of AgAu alloys), the surface diffusion of gold and silver atoms was captured by using *E*_*B*_^Au–Au^ = *E*_B_^Ag–Ag^ = 0.15 eV, and dissolution of silver atoms was captured using *E*_B_^Ag^ = 0.15 eV. In order to capture the mobility of Pt and Ir in this study, bond energies were scaled under the assumption that the activation energy for surface diffusion scales with the homologous temperature (within ±10%):^104^ *E*_B_^Pt–Pt^ = 0.23 eV, *E*_B_^Ir–Ir^ = 0.31 eV, *E*_B_^Ir–Pt^ = 0.19 eV, and *E*_B_^Au–Pt^ = 0.19 eV. The exponential prefactor, ν_1_ = 10^12^ s^–1^, is the surface Debye frequency for a Pt surface.^105,106^ In order to capture the activation energy for dissolution/redeposition of Au, Pt, and Ir in this study, the bond energy was scaled by their standard reduction potentials: *E*_B_^Pt–Pt^ = 0.19 eV, *E*_B_^Ir–Ir^ = 0.154 eV, *E*_B_^Au–Au^ = 0.22 eV, *E*_B_^Ir–Pt^ = 0.172 eV, and *E*_B_^Au–Pt^ = 0.205 eV. The exponential prefactor ν_2_ = 2 s^–1^ was chosen based on the exchange current density for Pt oxidation, in close agreement with a recent study by Erlebacher et al. on the evolution of Pt surfaces during cyclic voltammetry.

### Simulated Thermal Coarsening of Individual Nanoporous Nanoparticles

To study coarsening trends in np-NiPt nanoparticles, we first generated fully dense Ni_80_Pt_20_ nanoparticles, 80 atoms in diameter (∼22 nm in diameter). Simulations were initialized by placing atoms on a three-dimensional fcc lattice, and the type of atom (e.g., Pt or Ni) was assigned at random based on a weighted probability determined by the composition of the alloy (80 at. % Ni, 20 at. % Pt). Nanoporous particles were generated by dealloying Ni out of the fully dense particles under a constant potential of 1.2 eV at 300 K for a total of 10^4^ simulated seconds, or ∼10^8^ iterations. Figure S2 shows a simulated nanoparticle before and after dealloying; the nanoparticles have a slightly smaller diameter, ∼70–75 atoms (∼19 nm in diameter), in close agreement with the experimental np-NiPt particles. We generated 10 unique dealloyed nanoparticles, which were used as “seeds” for studying the impact of individual surface dopants on thermal and electrochemical coarsening. Dopants were introduced to the np-NiPt nanoparticles by depositing 3500 atoms (∼5 at. %) on the surface at locations with first nearest neighbor coordination numbers *n* ≤ 5; dopant deposition and density are visualized in Figure 2B of the main text.

A total of 30 porous nanoparticles (10 np-NiPt, 10 np-NiPt + Ir, and 10 np-NiPt + Au) underwent simulated thermal coarsening at 450 °C for 60 s. The dealloyed structure was output at regular intervals and analyzed using a hybrid meshing and fairing algorithm for topologically complex materials, outlined in ref 84. The hybrid method enables relevant structural features to be extracted from the simulations, such as the topological genus, interfacial shape distribution, surface area distribution, density of crystallographic facets, and surface defect density. For this study, we primarily focused on the surface area and facet distribution.

The data presented as bands in Figure 2 are a combination of the total surface area (the lower bound in Figure 3B) and an estimate of the active surface area (the upper bound in Figure 3B). The total surface area is the raw surface area calculated from every surface atom. The active surface area is the total surface area normalized by the area fraction of terrace atoms on (111) facets. This normalization accounts for integrated charge variations from *H*_upd_ adsorption due to alloy chemistry and facet distribution.^92^

### Simulated Electrochemical Coarsening of Individual Nanoporous Nanoparticles

Simulated electrochemical coarsening was carried out on the same 30 nanoparticles described above. Several kMC studies have implemented multistep oxidation/reduction mechanisms to capture the atomistic behavior of a Pt surface in acid and basic electrolytes during electrochemical cycling.^101,107^ These mechanisms are based on experimental LEED and STM observations, which reported a periodic length scale associated with roughening of Pt surfaces following Pt oxidation and reduction.^107,108^ This morphology is associated with a kinetic competition between surface roughening (oxide reduction) and surface smoothing from mobile Pt and Pt oxide species. We defined a potential-dependent “swapping” mechanism that encompasses atomic movement resulting from Pt oxidation/reduction. When this roughening event is chosen, the Pt atom is dissolved and redeposited at an unoccupied first nearest neighbor location. For the experimental condition of convection via electrolyte stirring, the roughening mechanism is modified to redeposit the dissolved Pt atom at a random, unoccupied surface site on the np-NiPt nanoparticle. An illustration of these mechanisms is shown in Figure S1. The simulated AST cycling conditions were chosen to mimic the experimental study, sweeping from ϕ = 0.6 to 1.1 eV at a ramp rate of 50 meV/s; the attempt frequencies and bond energies used are defined above.^31,46,109–111^

## Supplementary Material



## Data Availability

The data that support the findings of this study are available from the corresponding authors upon reasonable request.

## Notes

Particle TEM images; compositional data; kMC simulation outputs; performance data; and ICP-MS analysis results (PDF)

## References

[1] Seh Z. W., Kibsgaard J., Dickens C. F., Chorkendorff I., Nørskov J. K., Jaramillo T. F. (2017). Combining Theory and Experiment in
Electrocatalysis: Insights into Materials Design. Science.

[2] Chu S., Cui Y., Liu N. (2017). The Path towards
Sustainable Energy. Nat. Mater..

[3] Gasteiger H. A., Markovic N. M. (2009). Just a Dream-or Future Reality?. Science.

[4] Debe M. K. (2012). Electrocatalyst
Approaches and Challenges for Automotive Fuel Cells. Nature.

[5] Xie X., He C., Li B., He Y., Cullen D. A., Wegener E. C., Kropf A. J., Martinez U., Cheng Y., Engelhard M. H., Bowden M. E., Song M., Lemmon T., Li X. S., Nie Z., Liu J., Myers D. J., Zelenay P., Wang G., Wu G., Ramani V., Shao Y. (2020). Performance Enhancement and Degradation
Mechanism Identification of a Single-Atom Co-N-C Catalyst for Proton
Exchange Membrane Fuel Cells. Nat. Catal..

[6] Tian X., Lu X. F., Xia B. Y., Lou X. W. (2020). Advanced Electrocatalysts
for the Oxygen Reduction Reaction in Energy Conversion Technologies. Joule.

[7] Zhu J., Hu L., Zhao P., Lee L. Y. S., Wong K. Y. (2020). Recent Advances
in Electrocatalytic Hydrogen Evolution Using Nanoparticles. Chem. Rev..

[8] Mistry H., Varela A. S., Kühl S., Strasser P., Cuenya B. R. (2016). Nanostructured
Electrocatalysts with Tunable Activity and Selectivity. Nat. Rev. Mater..

[9] Nie Y., Li L., Wei Z. (2015). Recent Advancements
in Pt and Pt-Free Catalysts for
Oxygen Reduction Reaction. Chem. Soc. Rev..

[10] Shao M., Chang Q., Dodelet J., Chenitz R. (2016). Recent Advances in
Electrocatalysts for Oxygen Reduction Reaction. Chem. Rev..

[11] Huang X., Zhao Z., Cao L., Chen Y., Zhu E., Lin Z., Li M., Yan A., Zettl A., Wang Y. M., Duan X., Mueller T., Huang Y. (2015). High-Performance Transition
Metal - Doped Pt3Ni Octahedra for Oxygen Reduction Reaction. Science.

[12] Li Y., Van Cleve T., Sun R., Gawas R., Wang G., Tang M., Elabd Y. A., Snyder J., Neyerlin K. C. (2020). Modifying
the Electrocatalyst - Ionomer Interface via Sulfonated Poly­(Ionic
Liquid) Block Copolymers to Enable High- Performance Polymer Electrolyte
Fuel Cells. ACS Energy Lett..

[13] Li W., Liu J., Zhao D. (2016). Mesoporous Materials for Energy Conversion
and Storage
Devices. Nat. Rev. Mater..

[14] Tian X., Zhao X., Su Y. Q., Wang L., Wang H., Dang D., Chi B., Liu H., Hensen E. J. M., Lou X. W., Xia B. Y. (2019). Engineering Bunched Pt-Ni Alloy Nanocages
for Efficient Oxygen Reduction in Practical Fuel Cells. Science.

[15] Zhang L., Roling L., Wang X., Vara M., Chi M., Liu J., Choi S., Park J., Herron J., Xie Z., Mavrikakis M., Xia Y. (2015). Platinum-Based Nanocages with Subnanometer-Thick
Walls and Well-Defined, Controllable Facets. Science.

[16] Chen C., Kang Y., Huo Z., Zhu Z., Huang W., Xin H. L., Snyder J. D. (2014). Highly
Crystalline Multimetallic
Nanoframes with Three-Dimensional Electrocatalystic Surfaces. Science.

[17] Chattot R., Le Bacq O., Beermann V., Kühl S., Herranz J., Henning S., Kühn L., Asset T., Guétaz L., Renou G., Drnec J., Bordet P., Pasturel A., Eychmüller A., Schmidt T. J., Strasser P., Dubau L., Maillard F. (2018). Surface Distortion
as a Unifying Concept and Descriptor in Oxygen Reduction Reaction
Electrocatalysis. Nat. Mater..

[18] Snyder J., Fujita T., Chen M. W., Erlebacher J. (2010). Oxygen Reduction
in Nanoporous Metal-Ionic Liquid Composite Electrocatalysts. Nat. Mater..

[19] Snyder J., Livi K., Erlebacher J. (2013). Oxygen Reduction
Reaction Performance
of [MTBD]­[Beti]-Encapsulated Nanoporous NiPt Alloy Nanoparticles. Adv. Funct. Mater..

[20] Li M., Zhao Z., Cheng T., Fortunelli A., Chen C.-Y., Yu R., Zhang Q., Gu L., Merinov B. V., Lin Z., Zhu E., Yu T., Jia Q., Guo J., Zhang L., Goddard W. A., Huang Y., Duan X. (2016). Ultrafine Jagged Platinum Nanowires Enable Ultrahigh Mass Activity
for the Oxygen Reduction Reaction. Science.

[21] Lim B., Jiang M., Camargo P. H. C., Cho E. C., Tao J., Lu X., Zhu Y., Xia Y. (2009). Pd-Pt Bimetallic Nanodendrites with
High Activity for Oxygen Reduction. Science.

[22] Chen S., Li M., Gao M., Jin J., Van Spronsen M. A., Salmeron M. B., Yang P. (2020). High-Performance Pt-Co
Nanoframes
for Fuel-Cell Electrocatalysis. Nano Lett..

[23] Snyder J., McCue I., Livi K., Erlebacher J. (2012). Structure/Processing/Properties
Relationships in Nanoporous Nanoparticles As Applied to Catalysis
of the Cathodic Oxygen Reduction Reaction Structure/Processing/Properties
Relationships in Nanoporous Nanoparticles As Applied to Catalysis
of the Ca. J. Am. Chem. Soc..

[24] Lu Y., Du S., Steinberger-Wilckens R. (2016). Three-Dimensional Catalyst Electrodes
Based on PtPd Nanodendrites for Oxygen Reduction Reaction in PEFC
Applications. Appl. Catal., B.

[25] Dubau L., Asset T., Chattot R., Bonnaud C., Vanpeene V., Nelayah J., Maillard F. (2015). Tuning the
Performance and the Stability
of Porous Hollow PtNi/C Nanostructures for the Oxygen Reduction Reaction. ACS Catal..

[26] Kibsgaard J., Gorlin Y., Chen Z., Jaramillo T. (2012). Meso-Structured
Platinum Thin Films: Active and Stable Electrocatalysts for the Oxygen
Reduction Reaction. J. Am. Chem. Soc..

[27] Gan L., Heggen M., O’Malley R., Theobald B., Strasser P. (2013). Understanding
and Controlling Nanoporosity Formation for Improving the Stability
of Bimetallic Fuel Cell Catalysts. Nano Lett..

[28] Chen J., McLellan J., Siekkinen A., Xiong Y., Li Z., Xia Y. (2006). Facile Synthesis of Gold - Silver Nanocages with Controllable Pores
on the Surface. J. Am. Chem. Soc..

[29] Wang R., Higgins D., Prabhudev S., Lee D., Choi J., Hoque M., Botton G., Chen Z. (2015). Synthesis and Structural
Evolution of Pt Nanotubular Skeletons: Revealing the Source of Instability
for Nanostructured Electrocatalysts. J. Mater.
Chem. A.

[30] Park J., Kwon T., Kim J., Jin H., Kim H. Y., Kim B., Joo S. H., Lee K. (2018). Hollow Nanoparticles
as Emerging
Electrocatalysts for Renewable Energy Conversion Reactions. Chem. Soc. Rev..

[31] Li Y., Hart J. L., Taheri M. L., Snyder J. D. (2017). Morphological Instability
in Topologically Complex, Three-Dimensional Electrocatalytic Nanostructures. ACS Catal..

[32] Chen Y., Cheng T., Goddard W. A. (2020). Atomistic Explanation
of the Dramatically
Improved Oxygen Reduction Reaction of Jagged Platinum Nanowires, 50
Times Better than Pt. J. Am. Chem. Soc..

[33] Jauhar A. M., Hassan F. M., Cano Z. P., Hoque M. A., Chen Z. (2018). Platinum-Palladium
Core-Shell Nanoflower Catalyst with Improved Activity and Excellent
Durability for the Oxygen Reduction Reaction. Adv. Mater. Interfaces.

[34] Wang X., Figueroa-Cosme L., Yang X., Luo M., Liu J., Xie Z., Xia Y. (2016). Pt-Based Icosahedral Nanocages: Using a Combination
of {111} Facets, Twin Defects, and Ultrathin Walls to Greatly Enhance
Their Activity toward Oxygen Reduction. Nano
Lett..

[35] Chen S., Niu Z., Xie C., Gao M., Lai M., Li M., Yang P. (2018). Effects of Catalyst
Processing on the Activity and Stability of Pt-Ni
Nanoframe Electrocatalysts. ACS Nano.

[36] Jiang K., Zhao D., Guo S., Zhang X., Zhu X., Guo J., Lu G., Huang X. (2017). Efficient Oxygen Reduction
Catalysis
by Subnanometer Pt Alloy Nanowires. Sci. Adv..

[37] Lim J., Shin H., Kim M., Lee H., Lee K.-S., Kwon Y., Song D., Oh S., Kim H., Cho E. (2018). Ga-Doped Pt-Ni Octahedral Nanoparticles as a Highly Active and Durable
Electrocatalyst for Oxygen Reduction Reaction. Nano Lett..

[38] Guo W., Cheng L., Gao X., Xu J., Chen C., Liu P., He D., Tian L., Song J., Zhou H., Wu Y. (2023). Hierarchical Porous
Pt/ZrO_2_ Nanoframework for Efficient
Oxygen Reduction Reaction. ACS Catal..

[39] Huang X., Zhao Z., Cao L., Chen Y., Zhu E., Lin Z., Li M., Yan A., Zettl A., Wang Y. M., Duan X., Mueller T., Huang Y. (2015). High-Performance
Transition
Metal-Doped Pt_3_ Ni Octahedra for Oxygen Reduction Reaction. Science.

[40] Kim H. Y., Kwon T., Ha Y., Jun M., Baik H., Jeong H. Y., Kim H., Lee K., Joo S. H. (2020). Intermetallic
PtCu Nanoframes as Efficient Oxygen Reduction Electrocatalysts. Nano Lett..

[41] Xu G.-R., Wang B., Zhu J.-Y., Liu F.-Y., Chen Y., Zeng J.-H., Jiang J.-X., Liu Z.-H., Tang Y.-W., Lee J.-M. (2016). Morphological and
Interfacial Control of Platinum Nanostructures
for Electrocatalytic Oxygen Reduction. ACS Catal..

[42] Wang R., Xu C., Bi X., Ding Y. (2012). Nanoporous
Surface Alloys as Highly
Active and Durable Oxygen Reduction Reaction Electrocatalysts. Energy Environ. Sci..

[43] Zhao X., Chen S., Fang Z., Ding J., Sang W., Wang Y., Zhao J., Peng Z., Zeng J. (2015). Octahedral
Pd@Pt_1.8_ Ni Core-Shell Nanocrystals with Ultrathin PtNi
Alloy Shells as Active Catalysts for Oxygen Reduction Reaction. J. Am. Chem. Soc..

[44] Chen S., Zhao J., Su H., Li H., Wang H., Hu Z., Bao J., Zeng J. (2021). Pd-Pt Tesseracts
for the Oxygen Reduction
Reaction. J. Am. Chem. Soc..

[45] Wang X., Vara M., Luo M., Huang H., Ruditskiy A., Park J., Bao S., Liu J., Howe J., Chi M., Xie Z., Xia Y. (2015). Pd@Pt Core-Shell Concave Decahedra:
A Class of Catalysts for the Oxygen Reduction Reaction with Enhanced
Activity and Durability. J. Am. Chem. Soc..

[46] Bu L., Shao Q., E B., Guo J., Yao J., Huang X. (2017). PtPb/PtNi Intermetallic Core/Atomic Layer Shell Octahedra for Efficient
Oxygen Reduction Electrocatalysis. J. Am. Chem.
Soc..

[47] Beermann V., Gocyla M., Willinger E., Rudi S., Heggen M., Dunin-Borkowski R. E., Willinger M.-G., Strasser P. (2016). Rh-Doped Pt-Ni Octahedral
Nanoparticles: Understanding the Correlation between Elemental Distribution,
Oxygen Reduction Reaction, and Shape Stability. Nano Lett..

[48] Li H.-H., Ma S.-Y., Fu Q.-Q., Liu X.-J., Wu L., Yu S.-H. (2015). Scalable Bromide-Triggered
Synthesis of Pd@Pt Core-Shell Ultrathin
Nanowires with Enhanced Electrocatalytic Performance toward Oxygen
Reduction Reaction. J. Am. Chem. Soc..

[49] Baldizzone C., Gan L., Hodnik N., Keeley G. P., Kostka A., Heggen M., Strasser P., Mayrhofer K. J. J. (2015). Stability
of Dealloyed Porous Pt/Ni
Nanoparticles. ACS Catal..

[50] Luo M., Sun Y., Zhang X., Qin Y., Li M., Li Y., Li C., Yang Y., Wang L., Gao P., Lu G., Guo S. (2018). Stable High-Index Faceted Pt Skin on Zigzag-Like PtFe
Nanowires Enhances
Oxygen Reduction Catalysis. Adv. Mater..

[51] Zhou S., Xie M., Ding Y., Wang Z., Nguyen Q., Li K. K., Xia Y. (2024). Strain-Controlled Galvanic Synthesis of Platinum Icosahedral Nanoframes
and Their Enhanced Catalytic Activity toward Oxygen Reduction. Nano Lett..

[52] Chang F., Bai Z., Li M., Ren M., Liu T., Yang L., Zhong C.-J., Lu J. (2020). Strain-Modulated
Platinum-Palladium
Nanowires for Oxygen Reduction Reaction. Nano
Lett..

[53] Gong M., Xiao D., Deng Z., Zhang R., Xia W., Zhao T., Liu X., Shen T., Hu Y., Lu Y., Zhao X., Xin H., Wang D. (2021). Structure Evolution
of PtCu Nanoframes from Disordered to Ordered for the Oxygen Reduction
Reaction. Appl. Catal., B.

[54] Zhang N., Feng Y., Zhu X., Guo S., Guo J., Huang X. (2017). Superior Bifunctional Liquid Fuel Oxidation and Oxygen Reduction
Electrocatalysis Enabled by PtNiPd Core-Shell Nanowires. Adv. Mater..

[55] Chen Z., Waje M., Li W., Yan Y. (2007). Supportless Pt and
PtPd Nanotubes as Electrocatalysts for Oxygen-Reduction Reactions. Angew. Chem., Int. Ed..

[56] Liu H., Zhong P., Liu K., Han L., Zheng H., Yin Y., Gao C. (2018). Synthesis
of Ultrathin Platinum Nanoplates for Enhanced
Oxygen Reduction Activity. Chem. Sci..

[57] Yang T., Cao G., Huang Q., Ma Y., Wan S., Zhao H., Li N., Yin F., Sun X., Zhang D., Wang M. (2015). Truncated
Octahedral Platinum-Nickel-Iridium Ternary Electro-Catalyst for Oxygen
Reduction Reaction. J. Power Sources.

[58] Tu W., Chen K., Zhu L., Zai H., E B., Ke X., Chen C., Sui M., Chen Q., Li Y. (2019). Tungsten-Doping-Induced
Surface Reconstruction of Porous Ternary Pt-Based Alloy Electrocatalyst
for Oxygen Reduction. Adv. Funct. Mater..

[59] Kwon T., Jun M., Kim H. Y., Oh A., Park J., Baik H., Joo S. H., Lee K. (2018). Vertex-Reinforced PtCuCo Ternary
Nanoframes as Efficient and Stable Electrocatalysts for the Oxygen
Reduction Reaction and the Methanol Oxidation Reaction. Adv. Funct. Mater..

[60] US Drive US Drive Fuel Cell Technical Team Road Map; TRB, 2017.

[61] Paperzh K., Alekseenko A., Pankov I., Guterman V. (2024). Accelerated Stress
Tests for Pt/C Electrocatalysts: An Approach to Understanding the
Degradation Mechanisms. J. Electroanal. Chem..

[62] Li J., Xi Z., Pan Y.-T., Spendelow J. S., Duchesne P. N., Su D., Li Q., Yu C., Yin Z., Shen B., Kim Y. S., Zhang P., Sun S. (2018). Fe Stabilization by Intermetallic
L1_0_-FePt and Pt Catalysis Enhancement in L1_0_-FePt/Pt Nanoparticles for Efficient Oxygen Reduction Reaction in
Fuel Cells. J. Am. Chem. Soc..

[63] Hu Y., Jensen J. O., Bretzler P., Cleemann L. N., Yu J., Li Q. (2021). Revealing the Genuine Stability of the Reference Pt/C Electrocatalyst
toward the ORR. Electrochim. Acta.

[64] Imhof T., Della Bella R. K. F., Stühmeier B. M., Gasteiger H. A., Ledendecker M. (2023). Towards a Realistic Prediction of Catalyst Durability
from Liquid Half-Cell Tests. Phys. Chem. Chem.
Phys..

[65] Lopes P. P., Li D., Lv H., Wang C., Tripkovic D., Zhu Y., Schimmenti R., Daimon H., Kang Y., Snyder J., Becknell N., More K. L., Strmcnik D., Markovic N. M., Mavrikakis M., Stamenkovic V. R. (2020). Eliminating Dissolution of Platinum-Based
Electrocatalysts at the Atomic Scale. Nat. Mater..

[66] Beermann V., Holtz M. E., Padgett E., De Araujo J. F., Muller D. A., Strasser P. (2019). Real-Time Imaging of
Activation and
Degradation of Carbon Supported Octahedral Pt-Ni Alloy Fuel Cell Catalysts
at the Nanoscale Using: In Situ Electrochemical Liquid Cell STEM. Energy Environ. Sci..

[67] Zadick A., Dubau L., Sergent N., Berthomé G., Chatenet M. (2015). Huge Instability of Pt/C Catalysts
in Alkaline Medium. ACS Catal..

[68] Erlebacher J., Aziz M. J., Karma A., Dimitrov N., Sieradzki K. (2001). Evolution
of Nanoporosity in Dealloying. Nature.

[69] Alonso C., Salvarezza R. C., Vara J. M., Arvia a. J., Vazquez L., Bartolome a., Baro a. M. (1990). The Evaluation of Surface Diffusion
Coefficients of Gold and Platinum Atoms at Electrochemical Interfaces
from Combined STM-SEM Imaging and Electrochemical Techniques. J. Electrochem. Soc..

[70] Seebauer E. G., Allen C. E. (1995). Estimating Surface
Diffusion Coefficients. Prog. Surf. Sci..

[71] Bratsch S. G. (1989). Standard
Electrode Potentials and Temperature Coefficients in Water at 298.15
K. J. Phys. Chem. Ref. Data.

[72] Maier, S. A. Plasmonics: Fundamentals and Applications; Springer Science & Business Media, 2007; Vol. 677.

[73] Zhang Z., Shao C., Zou P., Zhang P., Zhang M., Mu J., Guo Z., Li X., Wang C., Liu Y. (2011). In Situ Assembly
of Well-Dispersed Gold Nanoparticles on Electrospun Silica Nanotubes
for Catalytic Reduction of 4-Nitrophenol. Chem.
Commun..

[74] Snyder J., Asanithi P., Dalton A. B., Erlebacher J. (2008). Stabilized
Nanoporous Metals by Dealloying Ternary Alloy Precursors. Adv. Mater..

[75] Erlebacher J. (2011). Mechanism
of Coarsening and Bubble Formation in High-Genus Nanoporous Metals. Phys. Rev. Lett..

[76] Granadillo L., Snyder J., Xia Z., McCue I. (2025). Coarsening Kinetics
of Alloy-Doped Nanoporous Metals. Scr. Mater..

[77] Pourbaix, M. Atlas of Electrochemical Equilibria in Aqueous Solutions; Pergamon Press: New York, 1966.

[78] Snyder J., Livi K., Erlebacher J. (2008). Dealloying
Silver/Gold Alloys in
Neutral Silver Nitrate Solution: Porosity Evolution, Surface Composition,
and Surface Oxides. J. Electrochem. Soc..

[79] Zhang J., Sasaki K., Sutter E., Adzic R. R. (2007). Stabilization of
Platinum Oxygen-Reduction Electrocatalysts Using Gold Clusters. Science.

[80] Gatalo M., Jovanovic P., Polymeros G., Grote J., Pavlisic A., Ruiz-Zepeda F., Selih V., Sala M., Hocevar S., Bele M., Mayrhofer K., Hodnik N., Gaberscek M. (2016). Positive Effect
of Surface Doping with Au on the Stability of Pt-Based Electrocatalysts. ACS Catal..

[81] Chen-Wiegart Y. C. K., Wang S., Chu Y. S., Liu W., McNulty I., Voorhees P. W., Dunand D. C. (2012). Structural Evolution
of Nanoporous
Gold during Thermal Coarsening. Acta Mater..

[82] Mendoza R., Thornton K., Savin I., Voorhees P. W. (2006). The Evolution of
Interfacial Topology during Coarsening. Acta
Mater..

[83] Lilleodden E. T., Voorhees P. W. (2018). On the Topological,
Morphological, and Microstructural
Characterization of Nanoporous Metals. MRS Bull..

[84] Erlebacher J., McCue I. (2012). Geometric Characterization of Nanoporous
Metals. Acta Mater..

[85] Mayrhofer K. J. J., Ashton S. J., Meier J. C., Wiberg G. K. H., Hanzlik M., Arenz M. (2008). Non-Destructive Transmission Electron
Microscopy Study of Catalyst
Degradation under Electrochemical Treatment. J. Power Sources.

[86] Sashikata K., Furuya N., Itaya K. (1991). In Situ Electrochemical
Scanning
Tunneling Microscopy of Single-Crystal Surfaces of Pt(111), Rh(111),
and Pd(111) in Aqueous Sulfuric Acid Solution. J. Vac. Sci. Technol., B:Microelectron. Nanometer Struct.--Process.,
Meas., Phenom..

[87] Jacobse L., Huang Y. F., Koper M. T. M., Rost M. J. (2018). Correlation of Surface
Site Formation to Nanoisland Growth in the Electrochemical Roughening
of Pt(111). Nat. Mater..

[88] Lopes P. P., Tripkovic D., Martins P. F. B. D., Strmcnik D., Ticianelli E. A., Stamenkovic V. R., Markovic N. M. (2018). Dynamics of Electrochemical Pt Dissolution
at Atomic and Molecular Levels. J. Electroanal.
Chem..

[89] Topalov A. A., Katsounaros I., Auinger M., Cherevko S., Meier J. C., Klemm S. O., Mayrhofer K. J. J. (2012). Dissolution of Platinum: Limits for
the Deployment of Electrochemical Energy Conversion?. Angew. Chem., Int. Ed..

[90] Cherevko S., Keeley G. P., Kulyk N., Mayrhofer K. J. J. (2016). Pt Sub-Monolayer
on Au: System Stability and Insights into Platinum Electrochemical
Dissolution. J. Electrochem. Soc..

[91] Xie X., Briega-Martos V., Alemany P., Mohandas
Sandhya A. L., Skála T., Rodríguez M. G., Nováková J., Dopita M., Vorochta M., Bruix A., Cherevko S., Neyman K. M., Matolínová I., Khalakhan I. (2025). Balancing
Activity and Stability through Compositional Engineering of Ternary
PtNi-Au Alloy ORR Catalysts. ACS Catal..

[92] van
der Vliet D. F., Wang C., Li D., Paulikas A. P., Greeley J., Rankin R. B., Strmcnik D., Tripkovic D., Markovic N. M., Stamenkovic V. R. (2012). Unique Electrochemical Adsorption
Properties of Pt-Skin Surfaces. Angew. Chem.,
Int. Ed..

[93] Brankovic S. R., Wang J. X., Adzic R. R. (2001). Metal Monolayer
Deposition by Replacement
of Metal Adlayers on Electrode Surfaces. Surf.
Sci..

[94] Cahan B. D., Villullas H. M. (1991). The Hanging
Meniscus Rotating Disk (HMRD). J. Electroanal.
Chem..

[95] Askew W. S., Beckmann R. B. (1965). Heat and Mass Transfer
in an Agitated Vessel. Ind. Eng. Chem. Process
Des. Dev..

[96] Wei Y. C., Liu C. W., Wang K. W. (2011). Improvement
of Oxygen Reduction Reaction
and Methanol Tolerance Characteristics for PdCo Electrocatalysts by
Au Alloying and CO Treatment. Chem. Commun..

[97] van
der Vliet D. F., Wang C., Tripkovic D., Strmcnik D., Zhang X. F., Debe M. K., Atanasoski R. T., Markovic N. M., Stamenkovic V. R. (2012). Mesostructured Thin Films as Electrocatalysts
with Tunable Composition and Surface Morphology. Nat. Mater..

[98] Lopes P. P., Strmcnik D., Tripkovic D., Connell J. G., Stamenkovic V., Markovic N. M. (2016). Relationships between
Atomic Level Surface Structure
and Stability/Activity of Platinum Surface Atoms in Aqueous Environments. ACS Catal..

[99] Erlebacher J. (2004). An Atomistic
Description of Dealloying. J. Electrochem. Soc..

[100] Erlebacher J., Aziz M. J., Karma A., Dimitrov N., Sieradzki K. (2001). Evolution
of Nanoporosity in Dealloying. Nature.

[101] Erlebacher J., Kubal J., Zeng Z., Greeley J., Struk K., Steinbach A. J. (2019). Kinetic Monte Carlo Simulations of
Electrochemical Oxidation and Reduction of Pt(111). J. Electrochem. Soc..

[102] McCue I., Snyder J., Li X., Chen Q., Sieradzki K., Erlebacher J. (2012). Apparent Inverse Gibbs-Thomson Effect
in Dealloyed Nanoporous Nanoparticles. Phys.
Rev. Lett..

[103] Li Y., Dinh Ngô B. N., Markmann J., Weissmüller J. (2019). Topology Evolution
during Coarsening of Nanoscale Metal Network Structures. Phys. Rev. Mater..

[104] Flynn C. P. (2006). Why Is Diffusion in Metals and on
Metal Surfaces Universal?. J. Phys.: Condens.
Matter.

[105] Winzer A. (1979). On the Quantum Theoretical Calculation
of Activation
Energies for the Self-Diffusion of Single Atoms and the Diffusion
of Adatoms on Metal Surfaces (I). Krist. Tech..

[106] Lyon H. B., Somorjai G. A. (1966). Surface Debye Temperatures of the
(100), (111), and (110) Faces of Platinum. J.
Chem. Phys..

[107] Harrington D. A. (1997). Simulation of Anodic Pt Oxide Growth. J. Electroanal. Chem..

[108] Fuchs T., Drnec J., Calle-Vallejo F., Stubb N., Sandbeck D. J. S., Ruge M., Cherevko S., Harrington D. A., Magnussen O. M. (2020). Structure Dependency of the Atomic-Scale
Mechanisms of Platinum Electro-Oxidation and Dissolution. Nat. Catal..

[109] Baldizzone C., Gan L., Hodnik N., Keeley G. P., Kostka A., Heggen M., Strasser P., Mayrhofer K. J. J. (2015). Stability
of Dealloyed Porous Pt/Ni Nanoparticles. ACS
Catal..

[110] Nie W., Tsai H., Asadpour R., Blancon J.-C., Neukirch A. J., Gupta G., Crochet J. J., Chhowalla M., Tretiak S., Alam M. A. (2015). High-Efficiency
Solution-Processed
Perovskite Solar Cells with Millimeter-Scale Grains. Science.

[111] Kwon H., Kabiraz M. K., Park J., Oh A., Baik H., Choi S. Il, Lee K. (2018). Dendrite-Embedded Platinum-Nickel
Multiframes as Highly Active and Durable Electrocatalyst toward the
Oxygen Reduction Reaction. Nano Lett..

