# Supplementary material for: Unveiling the Origin of Morphological Instability in Topologically Complex Electrocatalytic Nanostructures
Source: J Am Chem Soc. 2025 Sep 6;147(37):33482–94. doi: 10.1021/jacs.5c07842 (PMC12447482; doi:10.1021/jacs.5c07842)
Supplement: Supplementary file 1 [file ja5c07842_si_001.pdf]

Supplementary Materials for

Unveiling the Origin of Morphological Instability in Topologically Complex Electrocatalytic Nanostructures

Yawei Li[1,2], James L. Hart[3,4], Ramchandra Gawas[2], Zhiyong Xia[5], Pietro P. Lopes[6], Jieyu Zhang[1], Siming Li[1], Yucheng Wang[7], Mitra Taheri[4], Ian McCue[5,8*], Joshua Snyder[2*]

[1]School of Chemistry and Chemical Engineering, Shanxi University, Taiyuan 030006, China

[2]Department of Chemical Engineering, Drexel University, Philadelphia, PA 19104, USA

[3]Department of Material Science and Engineering, Drexel University, Philadelphia, PA 19014, USA

[4]Department of Materials Science and Engineering, Johns Hopkins University, Baltimore, MD 21218, USA

[5]Johns Hopkins University Applied Physics Laboratory, Laurel, MD 20723, USA

[6]Argonne National Laboratory, Materials Science Division, Lemont, IL 60439, USA

[7]College of Chemistry and Chemical Engineering, Xiamen University, Xiamen 361005, China

[8]Department of Materials Science and Engineering, Northwestern University, Easton, IL 60208, USA

**Corresponding authors: jds43@drexel.edu*, and *ian.mccue@jhuapl.edu*

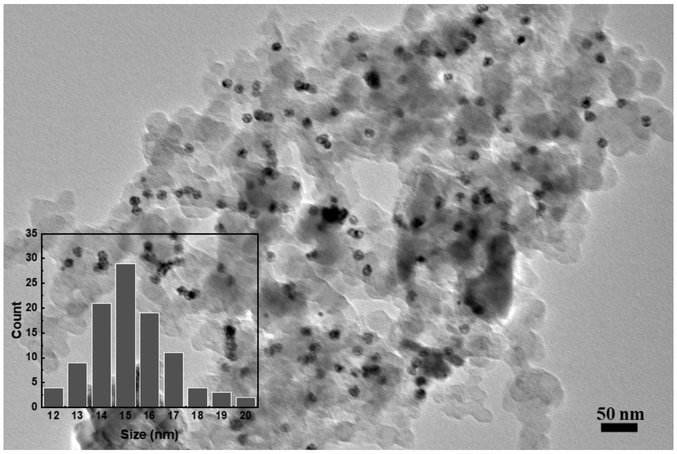

Figure S1. Representative TEM and size histogram for np-NiPt.

Table S1. Particle composition, both wt.% and at.%, from digestive ICP-MS for np-PtNi+Ir and np-PtNi+Au.

Sample	Pt	Ni	Ir/Au
np-PtNi+Ir	82.73 wt.%	10.82 wt.%	6.45 wt.%
np-PtNi+Au	82.93 wt.%	11.05 wt.%	6.02 wt.%
np-PtNi+Ir	66.06 at.%	28.71 at.%	5.23 at.%
np-PtNi+Au	66.02 at.%	29.23 at.%	4.75 at.%

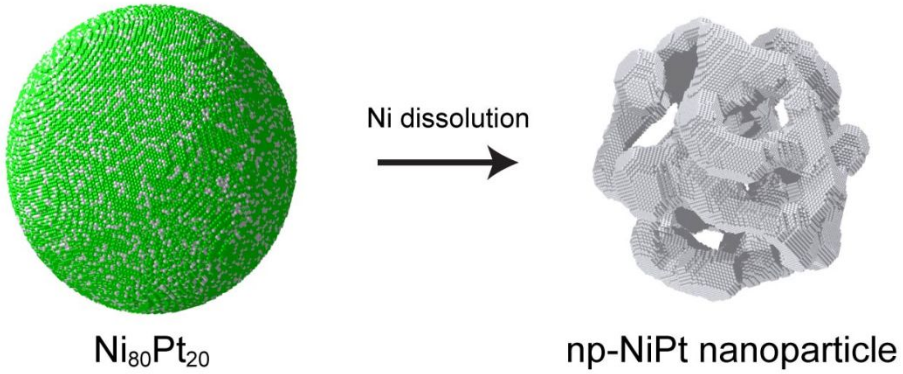

Figure S2. Illustration of NiPt nanoparticle as-synthesized (left) and after electrochemical dealloying (right). Green-colored atoms: Ni. Silver-colored atoms: Pt.

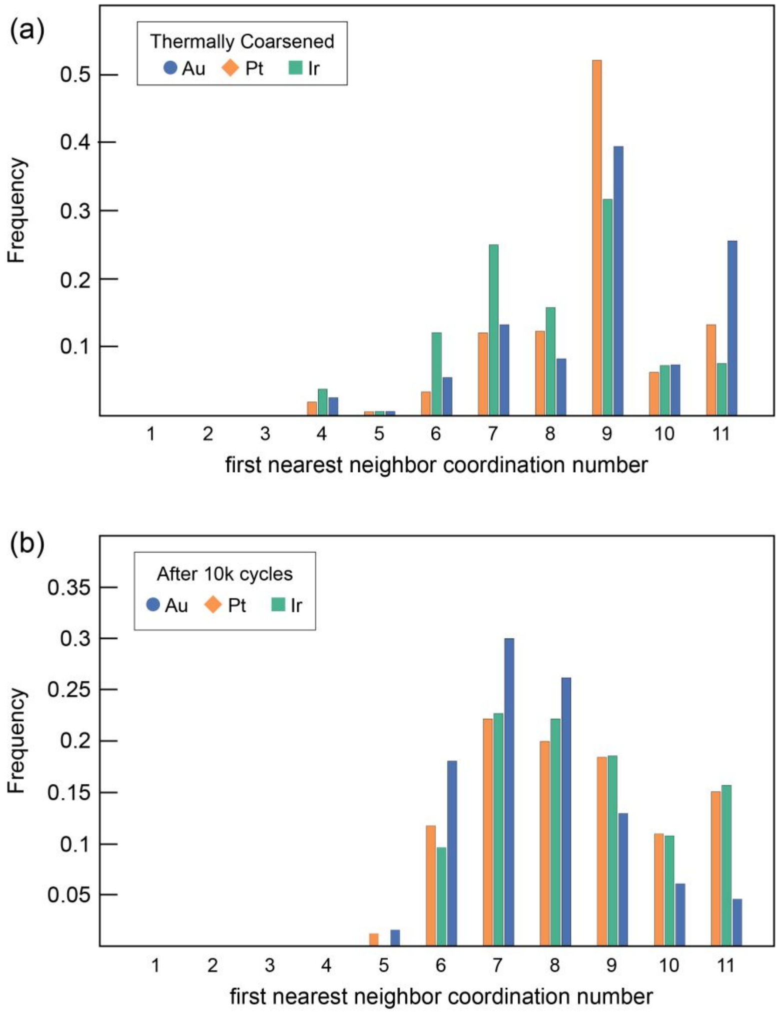

Figure S3. Surface defect density comparison of dopant species (Au, Pt, Ir) after simulated coarsening. (a) Surface defect density after thermal coarsening for 60 seconds at 400 ^{o}C. As the np-NiPt nanoparticles coarsen, there is an increase in the fraction of highly coordinated surface sites and decrease in step sites. However, owing to their sluggish surface mobility, there is a higher fraction of Ir dopants at six- and seven-coordinated sites. (b) Surface defect density after electrochemical coarsening for 10k cycles at room temperature. While each species has a preference to collect at six- and seven-coordinated sites, the fraction is substantially higher for Au dopants in comparison to Ir and Pt dopants.

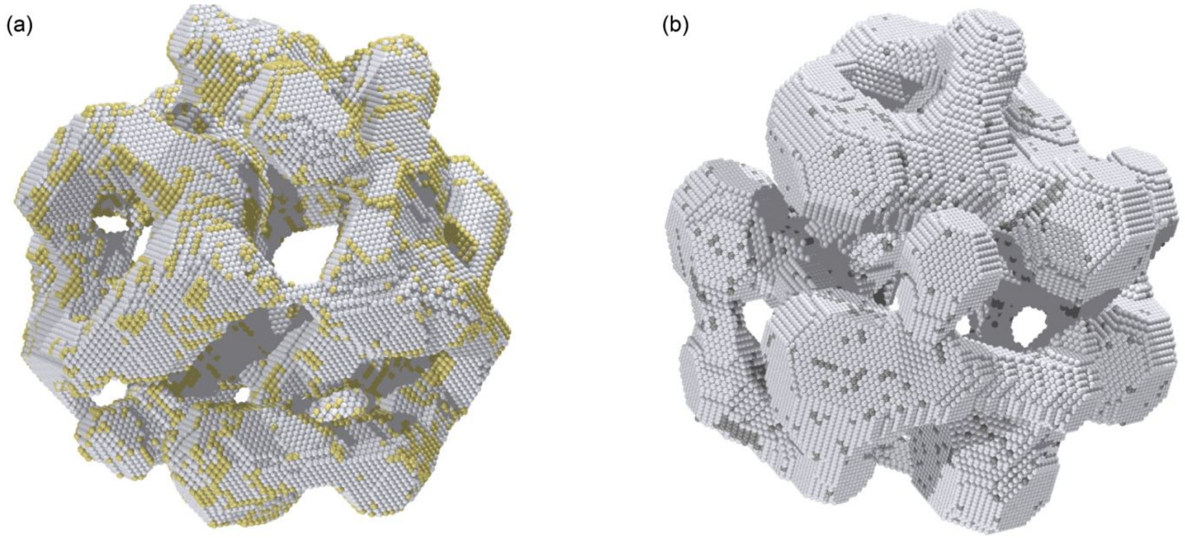

Figure S4. Representative images of (a) Au- and (b) Ir-doped nanoparticles after electrochemical coarsening for 10k cycles, under non-convective conditions. Images are full-size versions of those in Figure 3A in the main text.

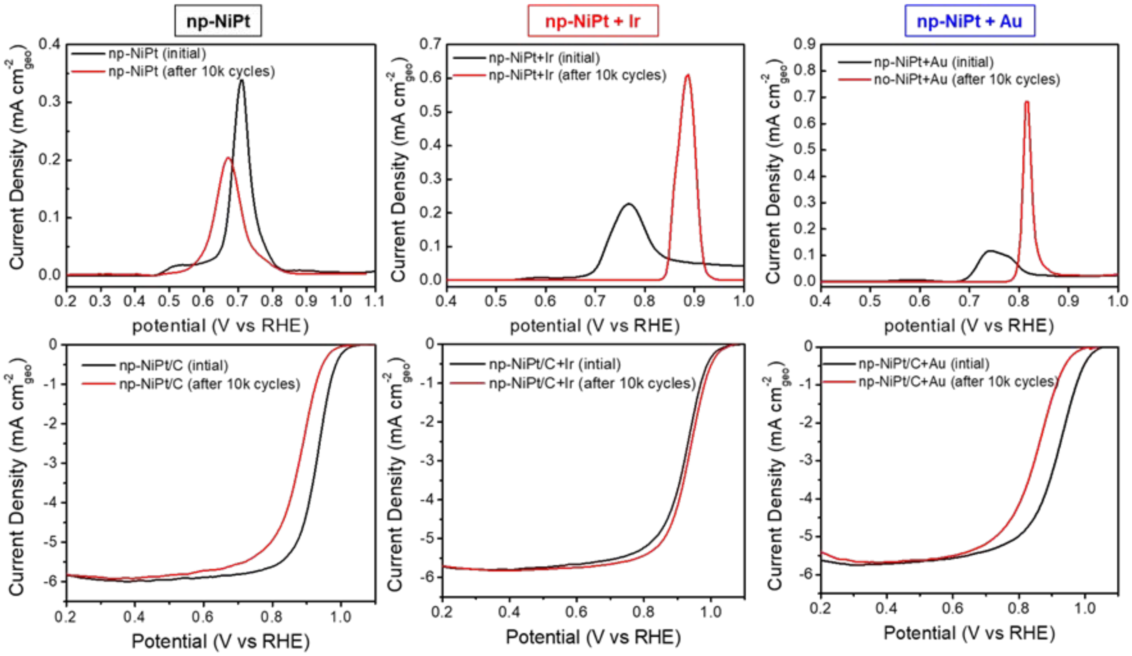

Figure S5. CO stripping (top) and ORR polarization (bottom) curves of np-NiPt, np-NiPt+Ir and np-NiPt+Au before (black) and after (red) AST in 0.1 M $HClO_4$ at room temperature with the UPL of 1.1 V vs RHE and a sweep rate of 50 mV s^{-1}.

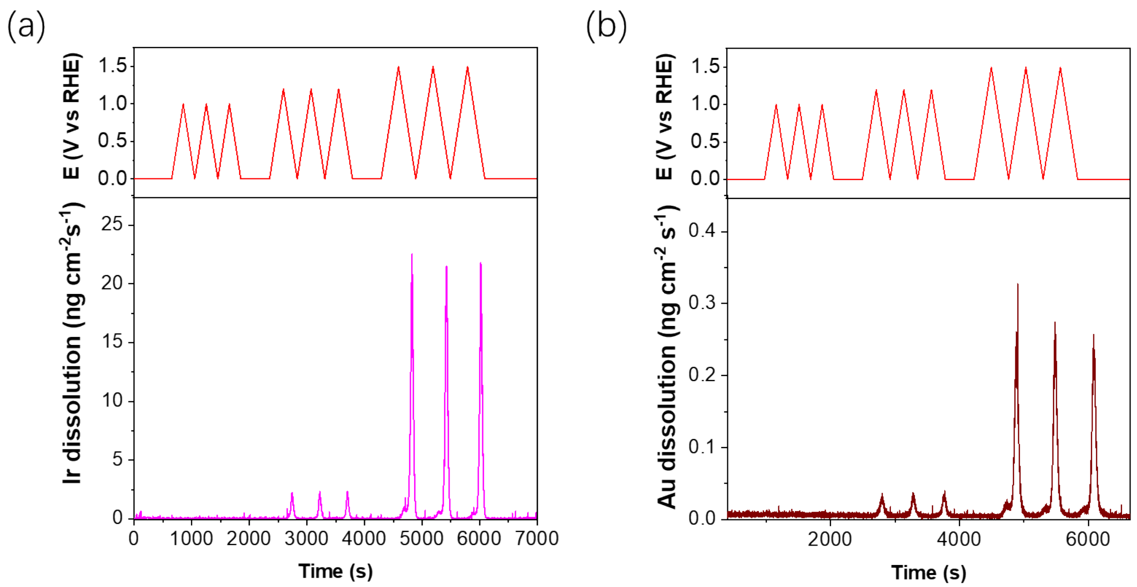

Figure S6. (a) Ir and (b) Au dissolution rates, measured with in-situ ICP-MS, for (a) np-NiPt+Ir and (b) np-NiPt+Au during triangular wave potential cycling for three upper potential limits 1.0, 1.2, and 1.5 V vs. RHE.

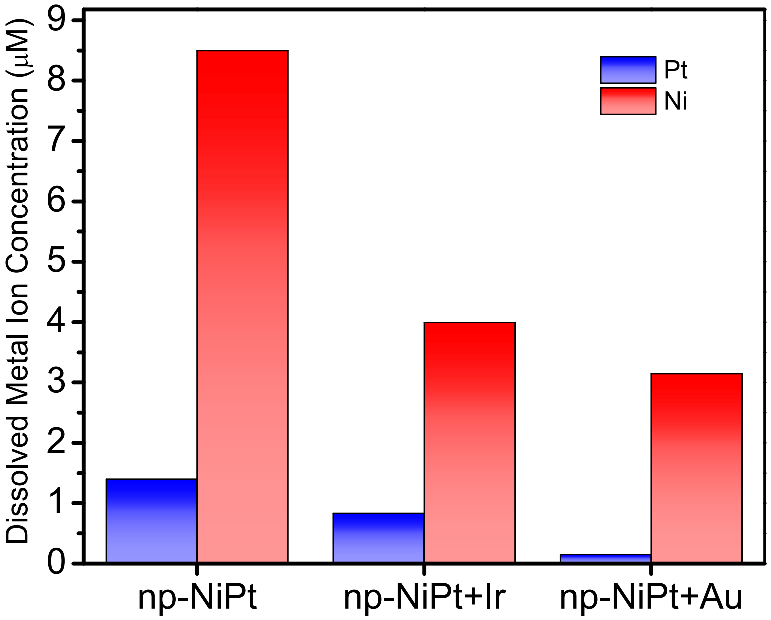

Figure S7. ICP-OES measurement of residual composition of dissolved Pt (blue) and Ni (red) in "aged" electrolyte for bare np-NiPt, np-NiPt+Ir and np-NiPt+Au after 10k AST potential cycles with UPL of 1.1 V vs. RHE.

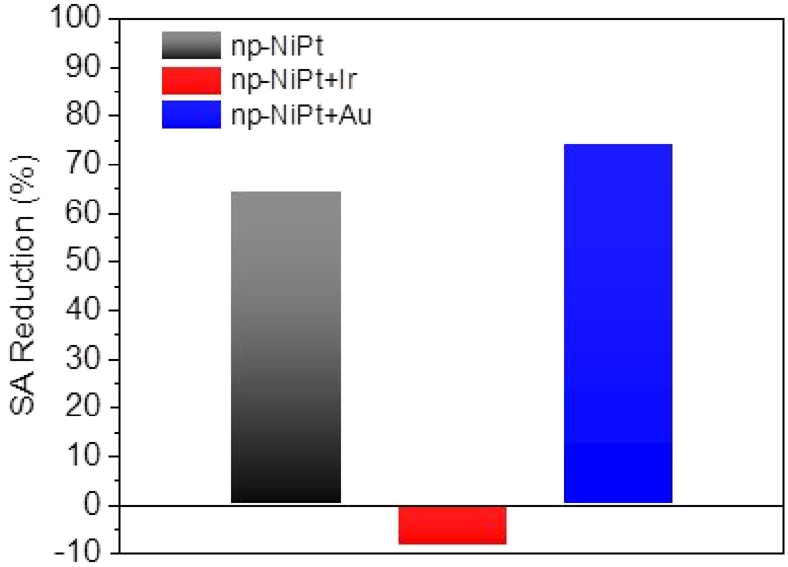

Figure S8. Average percent reduction in specific activity of bare np-NiPt, np-NiPt+Ir and np-NiPt+Au in O_2-saturated 0.1M $HClO_4$ after AST 10k cycles with UPL of 1.1 V vs RHE and sweep rate of 50 mV s^{-1} in Ar-saturated 0.1M $HClO_4$.

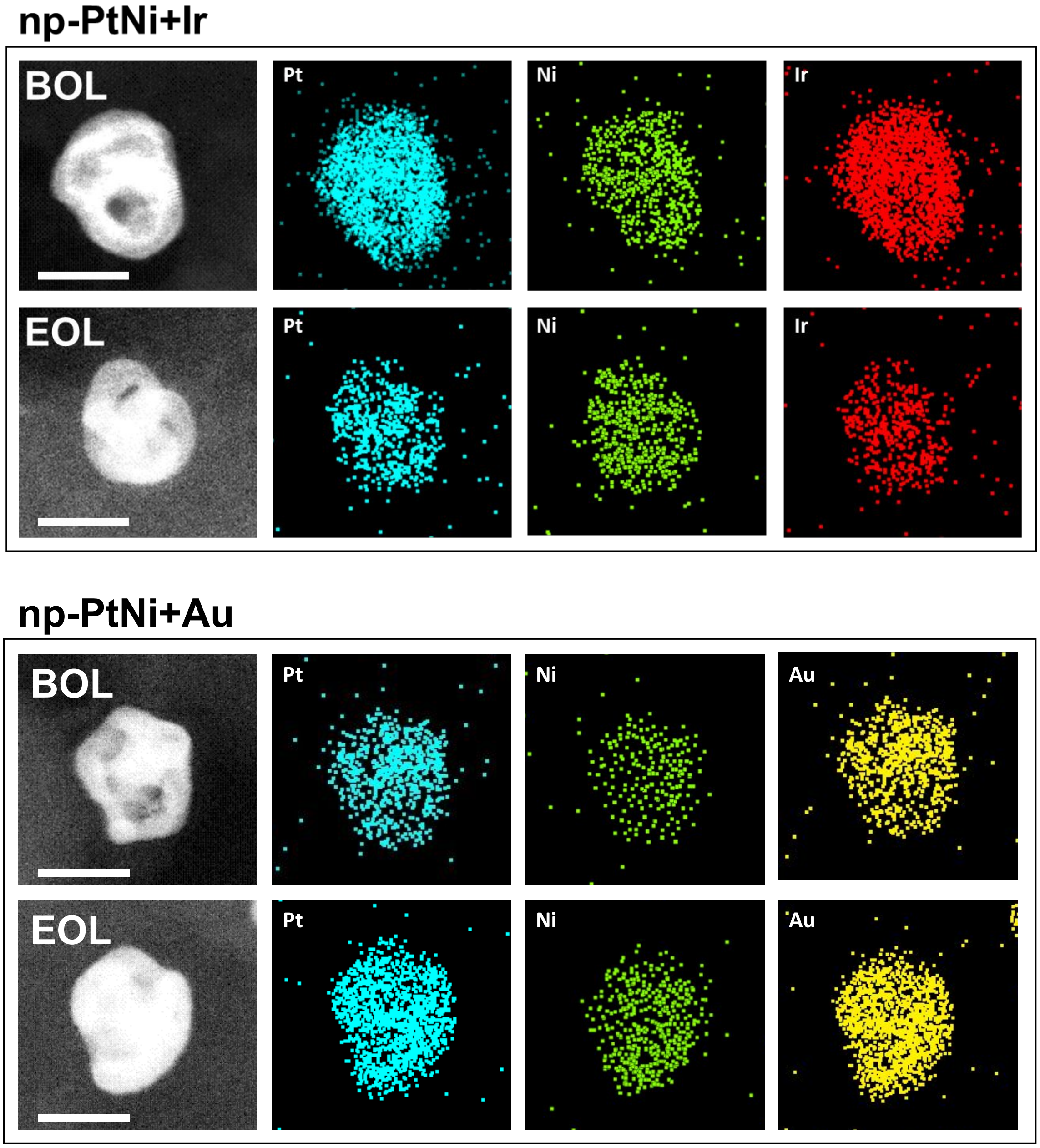

Figure S9: (Top) High resolution EDS mapping of Pt (blue), Ni (green), and Ir (red) for np-NiPt+Ir at beginning of life (BOL) (top row) and end of life (EOL) (bottom row) following 10,000 AST cycles up to 1.1 V vs. RHE. (Bottom) High resolution EDS mapping of Pt (blue), Ni (green), and Au (yellow) for np-NiPt+Au at beginning of life (BOL) (top row) and end of life (EOL) (bottom row) following 10,000 AST cycles up to 1.1 V vs. RHE.

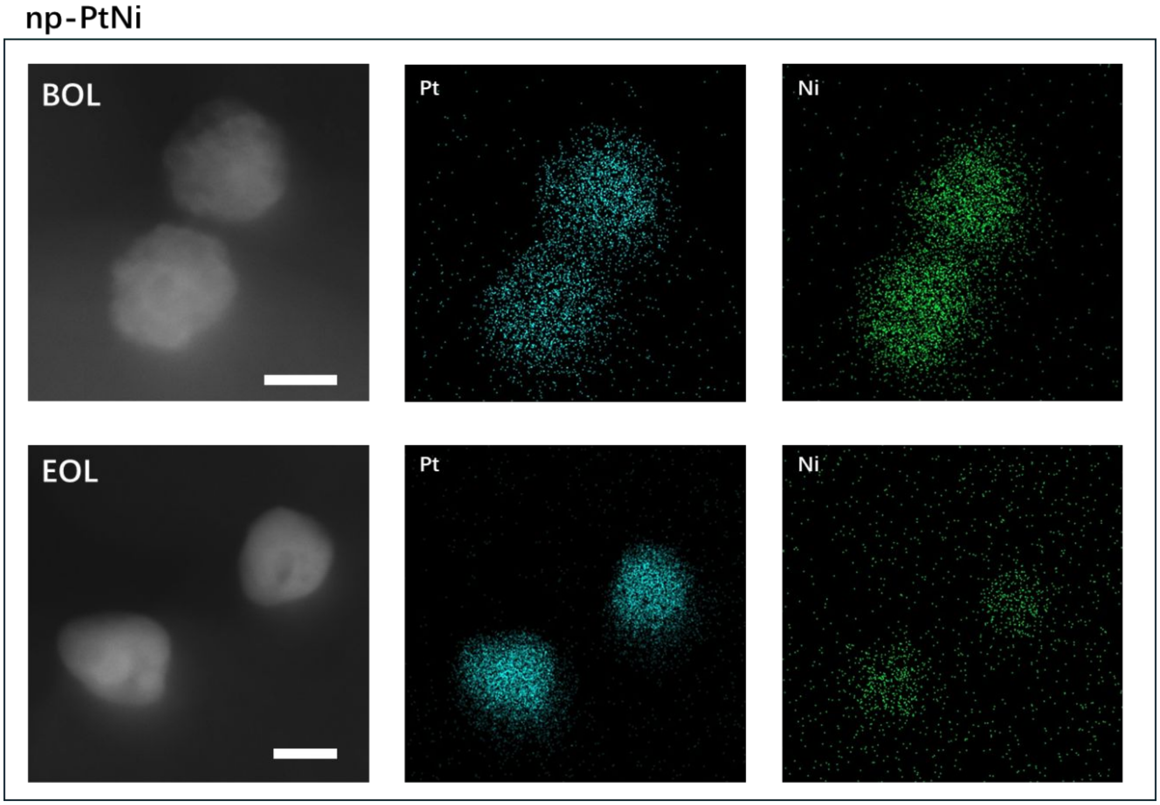

Figure S10. High resolution EDS mapping of Pt (green), and Ni (purple) for np-NiPt at beginning of life (BOL) (top row) and end of life (EOL) (bottom row) following 10,000 AST cycles up to 1.1 V vs. RHE.

Table S2. EDS quantification of Pt:Ni ratio for np-PtNi, np-PtNi+Ir and np-PtNi+Au at BOL and EOL.

atomic ratio of Pt:Ni	np-PtNi	np-PtNi+Ir	np-PtNi+Au
BOL	2.36: 1	2.48: 1	2.53: 1
EOL	9.87: 1	5.56: 1	3.85: 1

A brief discussion of the ORR trends:

The composition dependent trend in ORR specific/mass activity appears to differ from that observed for ECSA decay with AST cycles. Over the 10,000 AST cycles, the ORR activity of np-NiPt+Au is found to decay to a greater degree than that for np-NiPt. While the ORR activity for np-NiPt+Ir is found to increase over the course of the AST. We can explain this by thinking about the fate of the surface dopant species. The ICP-OES analysis detects minimal Au and Ir content eluted in solution during potential cycling. This is not surprising as both Au and Ir/IrO_x are known to be stable at the UPL of the AST testing. If the Ir and Au dopants are remaining on the particle surface while the surface area is slowly decreasing, the percentage of the surface of those particles that is covered in the dopant species is increasing. That is assuming that the dopant species are remaining on the surface and not incorporating deeper into the nanostructure. Consider the phase stability of dilute Au in Pt and dilute Ir in Pt. At the dilute range for Pt-Au, there is no Au solubility, meaning that the Au should remain on the surface of the nanostructure if it is not dissolving into solution, which ICP-OES indicates it is not. So as the particles are aging, the reduction in surface area would mean an increase in coverage of Au. This Au surface enrichment is confirmed by the TEM elemental mapping (Figure S7). If the surface has a higher Au coverage after the AST, it is understandable that the ORR activity would decrease even if the ECSA did not decrease dramatically. For np-NiPt+Ir, the ORR activity is found to increase after the AST over the initial ORR activity. This can be explained by looking at the Pt-Ir phase diagram. In contrast to Pt-Au, Ir has solubility in Pt in the dilute range. This means that Pt and Ir can alloy over the course of the AST. This alloying, even at room temperature, is promoted by the repetitive oxidation/reduction and dissolution/redeposition during the AST. TEM elemental mapping also indicates a slight reduction in the total Ir on the surface, likely due to limited loss during the AST protocol. The partial alloying of Ir with Pt at the surface of the catalyst along with a redistribution of Ir and coupled with the minimal drop in ECSA during the AST, results in a slight increase in ORR activity as compared to the beginning of life for the catalyst.
